# Hypoxia-activated probe for NIR fluorescence and photoacoustic dual-mode tumor imaging

**DOI:** 10.1016/j.isci.2021.102261

**Published:** 2021-03-02

**Authors:** Meng Li, Huan Li, Qian Wu, Niu Niu, Jiachang Huang, Lingmin Zhang, Ying Li, Dong Wang, Ben Zhong Tang

**Affiliations:** 1 Center for AIE Research, College of Materials Science and Engineering, Shenzhen University, Shenzhen 518060, P. R. China; 2 Key Laboratory of Optoelectronic Devices and Systems of Ministry of Education and Guangdong Province, College of Physics and Optoelectronic Engineering, Shenzhen University, Shenzhen 518060, P. R. China; 3 Department of Chemistry, Hong Kong Branch of Chinese National Engineering Research Center for Tissue Restoration and Reconstruction, The Hong Kong University of Science and Technology, Clear Water Bay, Kowloon, Hong Kong 999077, P. R. China; 4 Key Laboratory of Molecular Target & Clinical Pharmacology and the State Key Laboratory of Respiratory Disease, School of Pharmaceutical Sciences, Guangzhou Medical University, Guangzhou 511436, P. R. China

**Keywords:** Medical Imaging, Optical Imaging, Optical Property, Optical Materials

## Abstract

Construction of tumor microenvironment responsive probe with more than one imaging modality, in particular toward hypoxia of solid tumors, is an appealing yet significantly challenging task. In this work, we designed a hypoxia-activated probe TBTO (Triphenylamine-Benzothiadiazole-Triphenylamine derivative featuring four diethylamino N-Oxide groups) for *in vivo* imaging. TBTO could undergo bioreduction in a hypoxic microenvironment to yield compound TBT sharing both near-infrared (NIR) aggregation-induced emission and strong twisted intramolecular charge transfer features, which endows the probe with excellent performance in NIR fluorescence and photoacoustic dual-mode tumor imaging. This study offers useful insights into designing a new generation agent for clinical cancer diagnosis.

## Highlights

• Hypoxia-activable probe TBTO could be converted to TBT in reductive environment
• TBT exhibited red-shifted fluorescence and promoted photoacoustic signal
• TBTO was well-performed in fluorescence and photoacoustic dual-mode tumor imaging

## Graphical abstract

**Figure:**
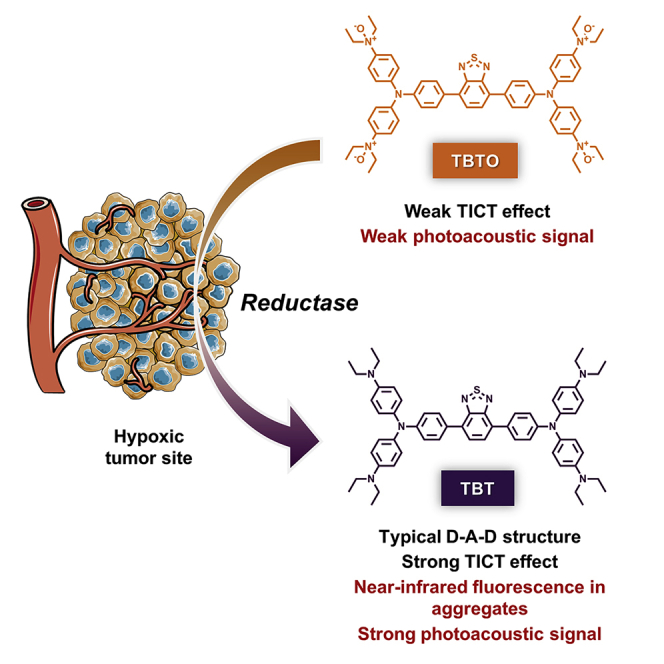

## Introduction

It is generally accepted that the rapid proliferation of tumor cells and abnormal vasculature in solid tumors could result in an inadequate supply of oxygen and thus lead to a hypoxic microenvironment, which is strongly associated with tumor propagation, malignant progression, and treatment resistance (Brown and Wilson, 2004; Harris, 2002; Rankin et al., 2016). Rapid and specific hypoxia detection *in vivo* is therefore of great importance in both scientific and clinical perspectives. On the other hand, hypoxia, as a symptom of a majority of solid tumors, can also serve as an internal stimulus to activate probes for specific tumor imaging with high contrast (Liu et al., 2017; Rey et al., 2017; Zhang et al., 2009).

Photoacoustic (PA) imaging is a rapidly emerging technique that provides three-dimensional information on the distribution of endogenous biomolecules using label-free PA imaging techniques (Hai et al., 2019; Wang et al., 2013; Wong et al., 2017; Yao et al., 2015) or exogenous PA probes in real-time noninvasively (Knox and Chan, 2018; Ou et al., 2019; Weber et al., 2016). Upon irradiation with a short-pulsed laser, the PA agents absorb the energy and partially convert it to heat, resulting in a local temperature increase and thermoelastic expansion. The pressure waves propagating through the surrounding tissue can be detected via ultrasound transducers. The ultrasound signal is much less scattered in biological tissue than the optical signal, which enables high-resolution PA imaging even at centimeter depths (Liu et al., 2016; Mallidi et al., 2011). Despite these merits, activatable PA agents, which show PA signal only in the presence of specific stimuli, have rarely been reported for *in vivo* applications (Knox et al., 2017; Miao et al., 2016; Roberts et al., 2018).

Twisted intramolecular charge transfer (TICT) is an electron transfer process that commonly occurs in molecules consisting of an electron donor-acceptor (D-A) pair linked by a single bond (Drummen, 2012; Sasaki et al., 2016). Upon photoexcitation, molecules in the TICT state returns to the ground state through red-shifted emission and enhanced nonradiative relaxation, which is usually accompanied by a strong PA signal output. The TICT effect can be easily tuned by modulation of substituents and configuration of D-A moieties, which makes the TICT effect an ideal strategy for activatable sensors (Hirayama et al., 2013; Reja et al., 2016; Ren et al., 2016). On the other hand, the aggregation-induced emission (AIE) phenomenon, a concept firstly coined in 2001 by Tang group, is another widely adopted strategy to design stimuli-responsive sensors since the optical properties of AIE luminogens (AIEgens) are potentially environment-dependent (Gao and Tang, 2017; Wang and Tang, 2019; Xu et al., 2020a). AIEgens commonly show enhanced emission in the aggregated state, which is mainly attributed to the restriction of intramolecular motions. AIEgens have made great contributions to bioimaging applications, especially those in the near-infrared (NIR) window, by regulating multi-hierarchical structures from single-molecule to molecular aggregates (Liu et al., 2020; Xu et al., 2020b; Zhang et al., 2020; Zhao et al., 2020). AIE effect endows molecules with high fluorescence efficiency while the TICT effect enables a red-shifted emission and enhanced non-radiative relaxation (Li et al., 2015, 2017). By molecular engineering in terms of adjusting the balance between TICT and AIE processes, fluorophores with NIR emission and excellent PA behavior can be obtained for NIR fluorescence and PA dual-mode imaging in deep tissue (Li et al., 2020c; Liu et al., 2019). However, the construction of stimuli-activated fluorophores that show turn-on NIR emission and PA signal triggered by the specific microenvironment *in vivo* is an appealing yet significantly challenging task (Li et al., 2018, 2020a; Meng et al., 2018).

Herein, we rationally designed and synthesized a hypoxia-responsive probe TBTO (Triphenylamine-Benzothiadiazole-Triphenylamine derivative featuring four diethylamino *N*-oxide groups) that could undergo bioreduction in a hypoxic microenvironment, producing TBT with a typical D-A-D structure. It was demonstrated that TBT possesses NIR fluorescence emission and PA signal generation, benefiting from its both AIE property and a strong TICT effect. *In vitro* and *in vivo* assessments revealed the responsiveness of TBTO in a reductive environment and its NIR fluorescence and PA dual-mode imaging ability.

## Results

### Design and synthesis of TBT and TBTO

The design of the hypoxia probe TBTO was based on previous reports demonstrating that the dimethylamino/diethylamino *N*-oxide group could be converted to dimethylamino/diethylamino group by reductases, such as cytochrome P450 (CYP450), over-produced in hypoxic regions of solid tumors (Albertella et al., 2008; Knox et al., 2017; Nishida et al., 2010). The synthetic route of TBTO is shown in Scheme S1 and all the products are fully characterized by nuclear magnetic resonance spectroscopy (NMR) and high-resolution mass spectra (See supplemental information for experimental details, Figures S1–S8). TBT with four diethylamino groups was firstly synthesized in a few steps with a high yield, and then the oxidation of TBT by *m*-chloroperbenzoic acid (*m*-CPBA) afforded TBTO having four diethylamino *N*-oxide groups.

**Scheme 1:**
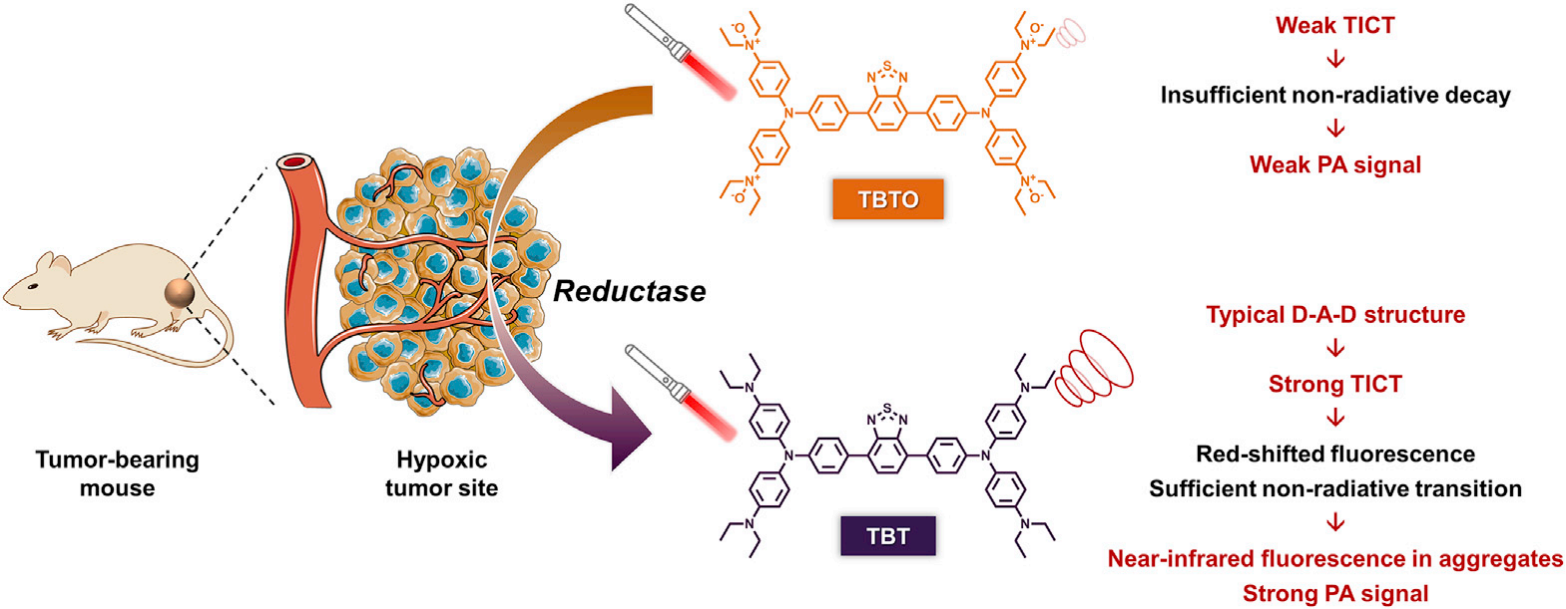
Schematic illustration of hypoxia-activated probe for NIR fluorescence and photoacoustic dual-mode tumor imaging Hypoxia probe TBTO undergoes reduction reaction by reductase, such as CYP450 enzymes in the hypoxic tumor site, to produce TBT, which shows red-shifted fluorescence and enhanced PA signal due to the strong TICT effect.

As illustrated in Scheme 1, we assume that TBTO could be converted to TBT by the reductases over-produced in the hypoxic tumor site. TBT has a typical D-A-D structure resulting from its inherent constituents including two triphenylamine fragments modified with diethylamino substituents acting as electron-donating units and a 2,1,3-benzothiadiazole group serving as an electron-accepting moiety. TICT is a common phenomenon in a molecule consisting of a D-A pair linked by a single bond (Scheme S2). In a polar environment such as the aqueous media in the biosystem, upon photoexcitation, such molecules undergo fast intramolecular electron transfer, accompanied by D-A twisting of the single bond. As a result, the locally excited state equilibrates rapidly with the TICT state with lower energy and subsequently returns to the ground state with a red-shifted emission. Additionally, the susceptibility of the TICT state favors multiple nonradiative quenching process, leading to a severely impaired fluorescence efficiency and enhanced PA signal output. In contrast, as for TBTO, the oxidation state of diethylamino decreases the electron-donating property of the nitrogen atom and prevents its lone pair from participating in the TPA conjugation system, leading to a much weaker TICT effect. Thus, TBTO would have a blue-shifted fluorescence emission and neglectable PA signal (Scheme 1).

### Photophysical properties of TBT and TBTO

**Figure 1:**
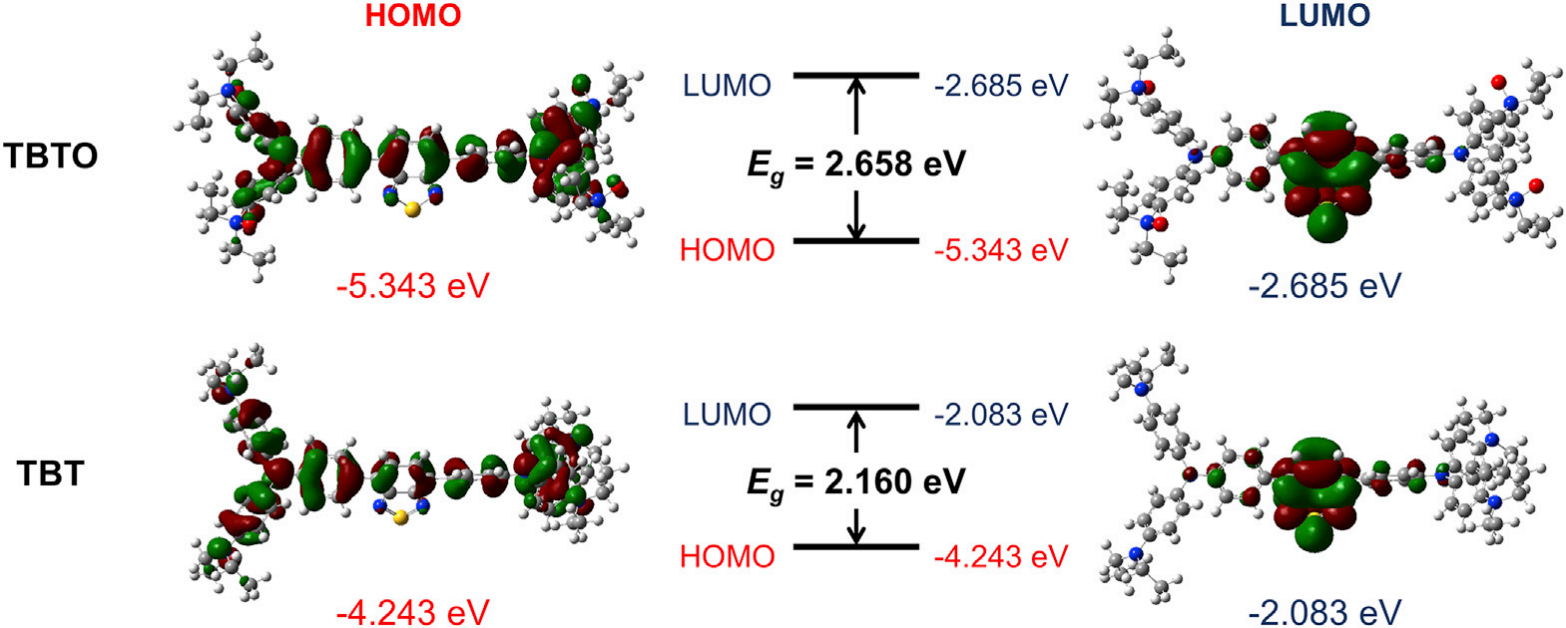
Density functional theory (DFT) calculations Molecular orbital amplitude plots of the highest occupied molecular orbital (HOMO) and lowest unoccupied molecular orbital (LUMO) energy levels of TBTO and TBO calculated at the B3LYP/6-31+G(d) level.

The introduction of electron-donating groups to a D-A effect featured fluorophore usually leads to red-shifted absorption and fluorescence emission. Indeed, as shown in Table 1, TBTO had an absorption maximum at 450 nm in tetrahydrofuran (THF) solution while the absorption of TBT centered at 530 nm. In the solid state, the emission maxima of TBTO and TBT were determined to be 600 and 740 nm, respectively. To have a better understanding of the above phenomenon, density functional theory (DFT) calculations were conducted at the B3LYP/6-31+G(d) level (Figure 1). Due to the strong electron-donating ability of the diethylamino group as compared to its oxidized form, the frontier molecular orbitals (both HOMO and LUMO) of TBT are more delocalized as compared to those of TBTO, leading to elevated HOMO and LUMO energy levels (Mao et al., 2018). Moreover, TBT possesses a much smaller HOMO-LUMO energy gap than TBTO, which is well consistent with the experimental data depicted in Table 1 that TBT has a red-shifted absorption and emission.

**Table 1:** Photophysical properties of TBTO and TBT

Compound	ε (M^−1^·cm^−1^)^a^	λ_abs, max_^a^	λ_em, max_^a^	λ_em, max_^b^	QY^a^	QY^b^
**TBTO**	0.7×10^4^	450 nm	592 nm	600 nm	69%	8.2%
**TBT**	1.8×10^4^	530 nm	Undetectable	740 nm	0.2%	5.7%

a in THF solution.

b in the solid state.

**Figure 2:**
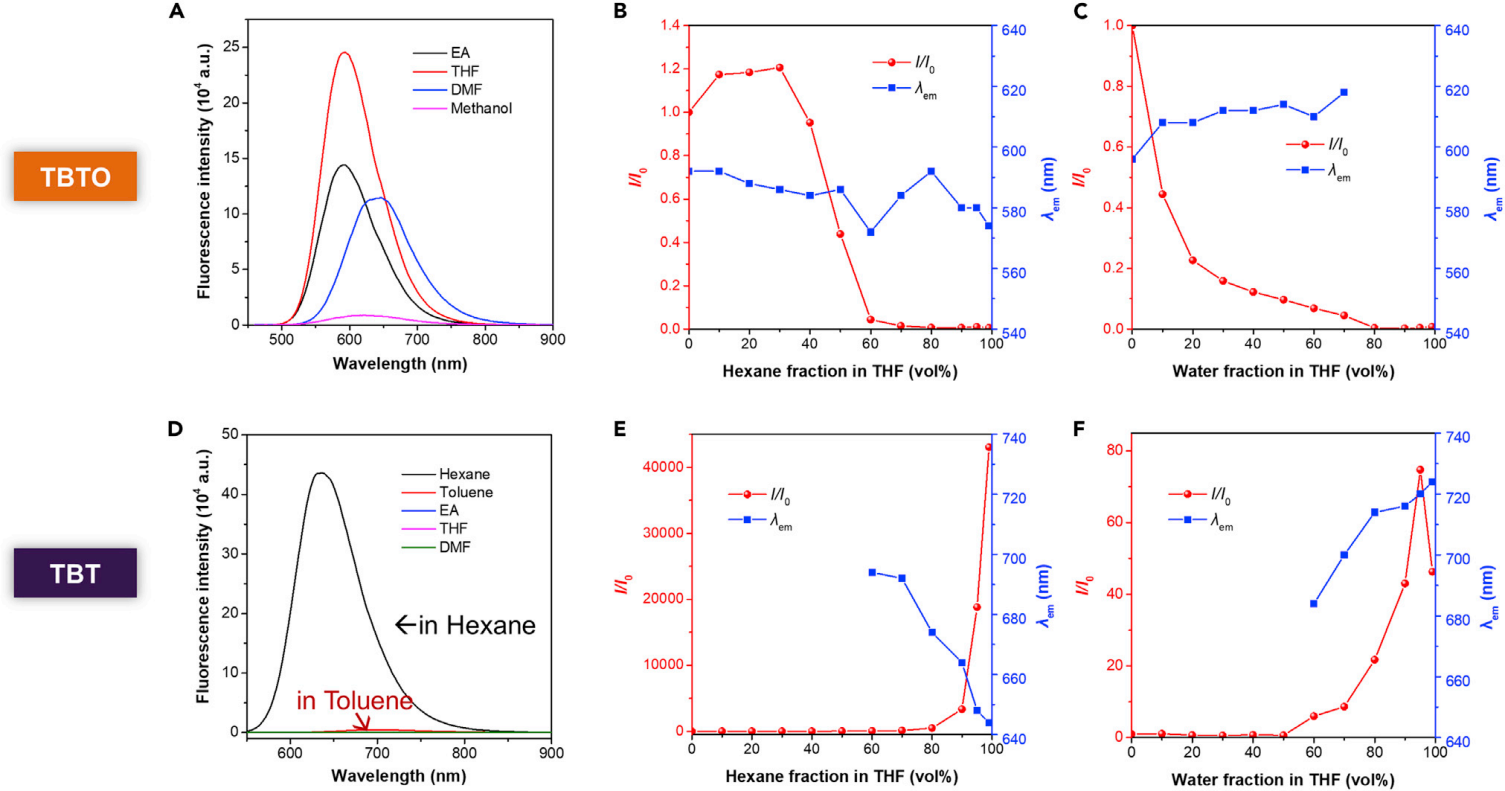
Solvatochromic effect of TBT and TBTO in different solvents (A and D) Fluorescence emission spectra of (A) TBTO and (D) TBT in different solvents. [TBTO], [TBT] = 20 μM. (B and E) Fluorescence emission changes of (B) TBTO and (E) TBT in THF with different fractions of hexane (red curve: the fluorescence intensity ratio *I/I*_*0*_ in the function of hexane fraction, *I*_*0*_ indicates the fluorescence intensity in pure THF; blue curve: the maximum emission wavelength in the function of hexane fraction). [TBTO], [TBT] = 20 μM. (C and F) Fluorescence emission changes of (C) TBTO and (F) TBT in THF with different fractions of water (red curve: the fluorescence intensity ratio *I/I*_*0*_ in the function of water fraction, *I*_*0*_ indicates the fluorescence intensity in pure THF; blue curve: the maximum emission wavelength in the function of water fraction). [TBTO], [TBT] = 20 μM.

To compare their TICT behavior, we evaluated the solvatochromic property of each compound by using a variety of solvents including two non-polar solvents (hexane and toluene), two less polar solvents (THF and ethyl acetate), and two polar solvents (dimethylformamide and methanol) (polarity: hexane < toluene << THF ∼ ethyl acetate < dimethylformamide < methanol). As depicted in Figure 2D, TBT was only emissive in hexane and toluene, and the fluorescence in polar solvents is not detectable under the same measurement conditions. Besides, the maximum emission of TBT red shifted from 637 nm to 700 nm when changing the solvent from hexane to toluene (Figure S9D). The obvious fluorescence quenching and red-shift with the increasing solvent polarity solidly indicated the strong TICT effect of TBT. It's worth mentioning that there is no obvious shift of the absorption spectra of TBT in different solvents (Figure S9C), suggesting that the above solvatochromic behavior was not caused by the difference of light-harvesting. Since TBTO is not soluble in hexane and toluene, the fluorescence emission spectra in the other four solvents were recorded. As displayed in Figures 2A and S9B, TBTO was emissive in all the polar solvents. Although the fluorescence changes of TBTO were observed in terms of both emission wavelength and intensity upon altering solvent polarity, the tendency was not accordant with the expected outcomes of the TICT effect, presenting the non-TICT feature of TBTO.

Furthermore, the solvatochromic behavior of those two compounds was investigated in mixed solvent systems. In the case of THF-hexane mixtures, TBT showed gradually blue-shifted and enhanced fluorescence with the increasing of the fraction of hexane (Figures 2E and S10C); while TBTO exhibit a decreased emission with little shift (Figures 2B and S10A). In the case of THF-water mixtures, similar results were observed for TBTO (Figures 2C and S10B); while TBT displayed gradually red-shifted emission with the addition of water as a strong polar solvent (Figures 2F and S10D). These results are in full agreement with our assumption that TBT is a typical TICT molecule whereas TBTO is not. It is important to note that the fluorescence of TBT gradually enhanced with the increasing of the fraction of water, which could be ascribed to the AIE property of TBT. The fluorescence quantum yield (QY) values of TBT in THF solution (Φ_FL_ = 0.2%) and the solid state (Φ_FL_ = 5.7%) provided additional evidence for the AIE behavior of TBT (Table 1).

To gain insights into the non-radiative relaxation efficiency of TBT and TBTO, the photothermal conversion performance was estimated under the irradiation of a 660 nm laser at a power of 0.5 W/cm^2^. As a result, the temperature of TBT solution increased by 20°C within 3 min irradiation, whereas the TBTO solution maintained almost the same temperature (Figure S11). This revealed that TBT possesses a relatively high non-radiative relaxation efficiency, which is favorable for PA signal generation. Importantly, TBT has a higher molar extinction efficiency than TBTO (Table 1), which is also beneficial for PA imaging according to the literature reported (Borg and Rochford, 2018). All these results suggested the significant capacity of TBTO for potential PA and fluorescence dual-mode imaging, once exposed to a reductive environment.

### Preparation and characterization of TBTO NPs and TBT NPs

**Figure 3:**
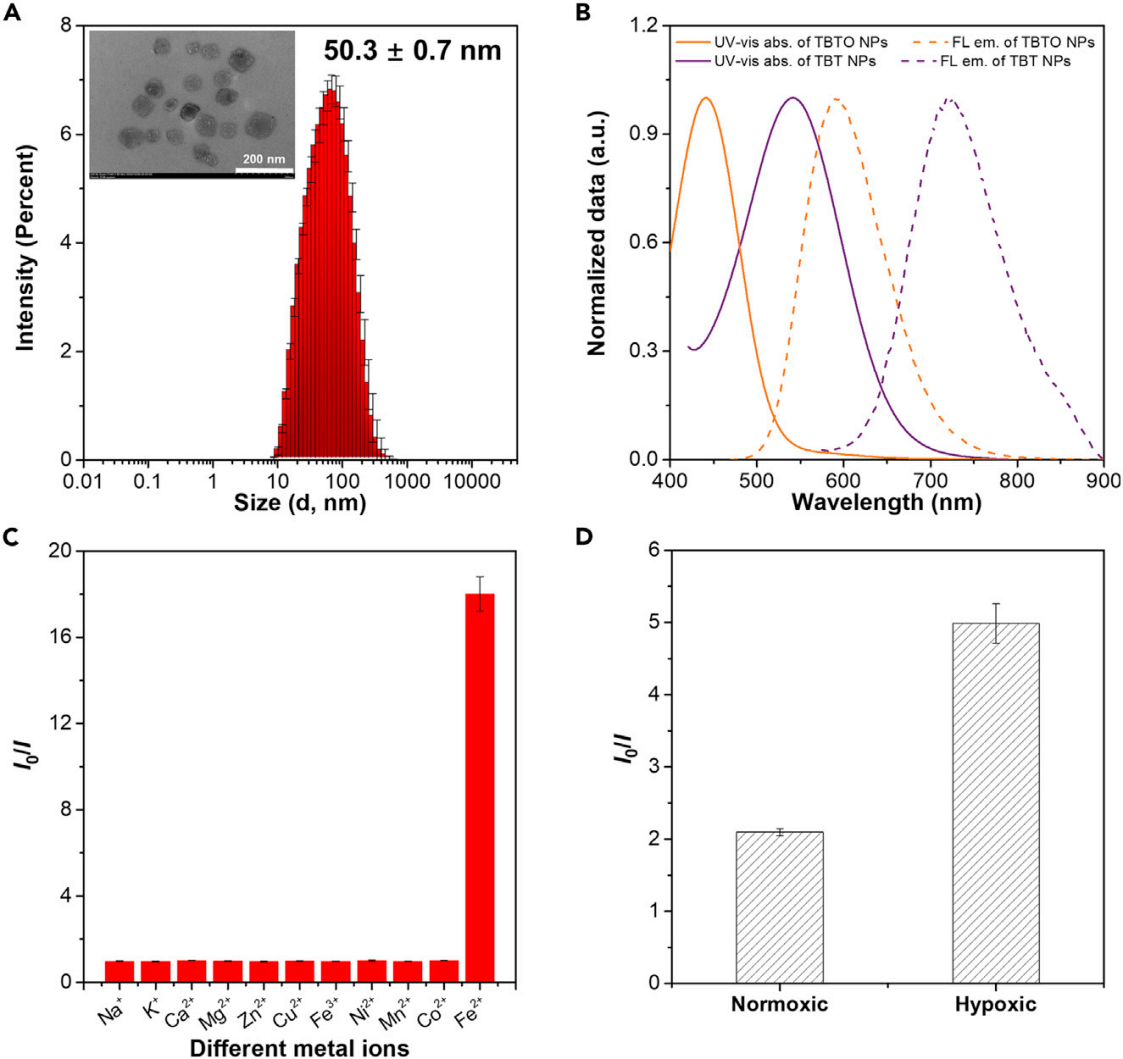
Characterization of TBTO NPs and TBT NPs, and *in vitro* responsiveness of TBTO NPs to reductive environment (A) Size distribution of TBTO NPs. Insert picture: TEM image of TBTO NPs. Data are represented as mean ± SD from three replicates. (B) Normalized absorption and emission spectra of TBTO NPs and TBT NPs. (C) Fluorescence responses of TBTO NPs (50 μM) to different metal ions (500 μM) in PBS buffer (pH 7.4) after 30 min incubation. Data are represented as mean ± SD from three replicates. (D) Fluorescence responses of TBTO NPs to different culture conditions (normoxic or hypoxic) of HeLa cells. Data are represented as mean ± SD from three replicates.

Before testing the responsibility of TBTO to the reductive species, TBTO nanoparticles (TBTO NPs) were fabricated to improve the biocompatibility and dispersibility in aqueous solution by encapsulating TBTO with an amphiphilic co-polymer DSPE-mPEG2000 through a typical nanoprecipitation method. Dynamic light scattering measurement revealed that the average size of TBTO NPs is around 50 nm, which is in agreement with the transmission electron microscopy (TEM) image (Figure 3A, Table S1). Additionally, the Zeta potential of TBTO NPs was measured to be −23 mV (Table S1), and the negatively charged NPs are favorable for long blood circulation and resistant to non-specific interactions for *in vivo* analysis. TBTO NPs have a maximum absorption at 441 nm and maximum emission at 596 nm (Figure 3B). Moreover, TBT NPs were also prepared using the same protocol as above presenting an average size of 103 nm and Zeta potential of −41.2 mV (Figure S12, Table S1). TBT NPs have a maximum absorption at 542 nm and maximum emission at 724 nm (Figure 3B). The spectral separation of TBTO NPs and TBT NPs allows for selective detection of a signal from one compound without apparent interference from the other.

### Responsibility of TBTO NPs to the reductive environment *in vitro*

Diethylamino *N*-oxide groups have previously been proven to be reduced by Fe(II), thus it is critical to determine the response of TBTO NPs to Fe(II) among different metal ions (Hirayama et al., 2013, 2017; Knox et al., 2017; Xu et al., 2019). To that end, TBTO NPs were incubated with various metal ions in PBS at 37°C and the fluorescence intensity at 600 nm with an excitation of 450 nm was measured every 5 min to monitor the consumption of TBTO (Figure S13). The fluorescence emission of TBTO was strongly quenched by 18 times with prolonging the incubation time to 30 min in the presence of 10 equivalents of Fe(II) (Figure 3C). As for other metal ions, no fluorescence change was detected. Furthermore, totally different optical spectra of TBTO NPs solution were observed with or without Fe(II) treatment (Figure S14), strongly suggesting the specific responsiveness of TBTO to reductive species. The transition of TBTO to TBT was verified by ^1^H-NMR using Na_2_SO_3_ as the reductive agent (Figure S15).

We then sought to determine whether TBTO NPs could undergo the hypoxia-responsive fluorescence quenching at a cellular level before moving to *in vivo* studies. HeLa cells, a human cervical cancer cell line, were firstly cultured either under a standard atmosphere containing 20% oxygen (normoxic condition) or in a sealed container with an anaerobic gas generator to keep the oxygen level lower than 0.1% (hypoxic condition). After pre-incubation for 12 h in the above conditions, cells were treated with 50 μM TBTO NPs and then a further incubation for 3 h under normoxic or hypoxic conditions. After that, the fluorescence intensities at 600 nm (*λ*_ex_: 450 nm) of the cell culture supernatants were measured in a microplate reader. As compared to the normoxic conditions, TBTO NPs suffered from a much stronger fluorescence quenching in the cells cultured in hypoxic conditions (Figure 3D). These outcomes revealed the efficient responsiveness of TBTO NPs to the reductive environment of cells cultured under hypoxia. The colocalization experiments performed with different organelle trackers indicated that TBTO NPs were mostly located in lipid droplets (Figure S16). It is worth mentioning that neither TBTO nor TBT exhibited cytotoxicity to HeLa cells at a concentration up to 100 μM indicating the biocompatibility of TBTO as a hypoxia probe (Figure S17).

### Hypoxia-responsive NIR fluorescence imaging of TBTO NPs *in vivo*

**Figure 4:**
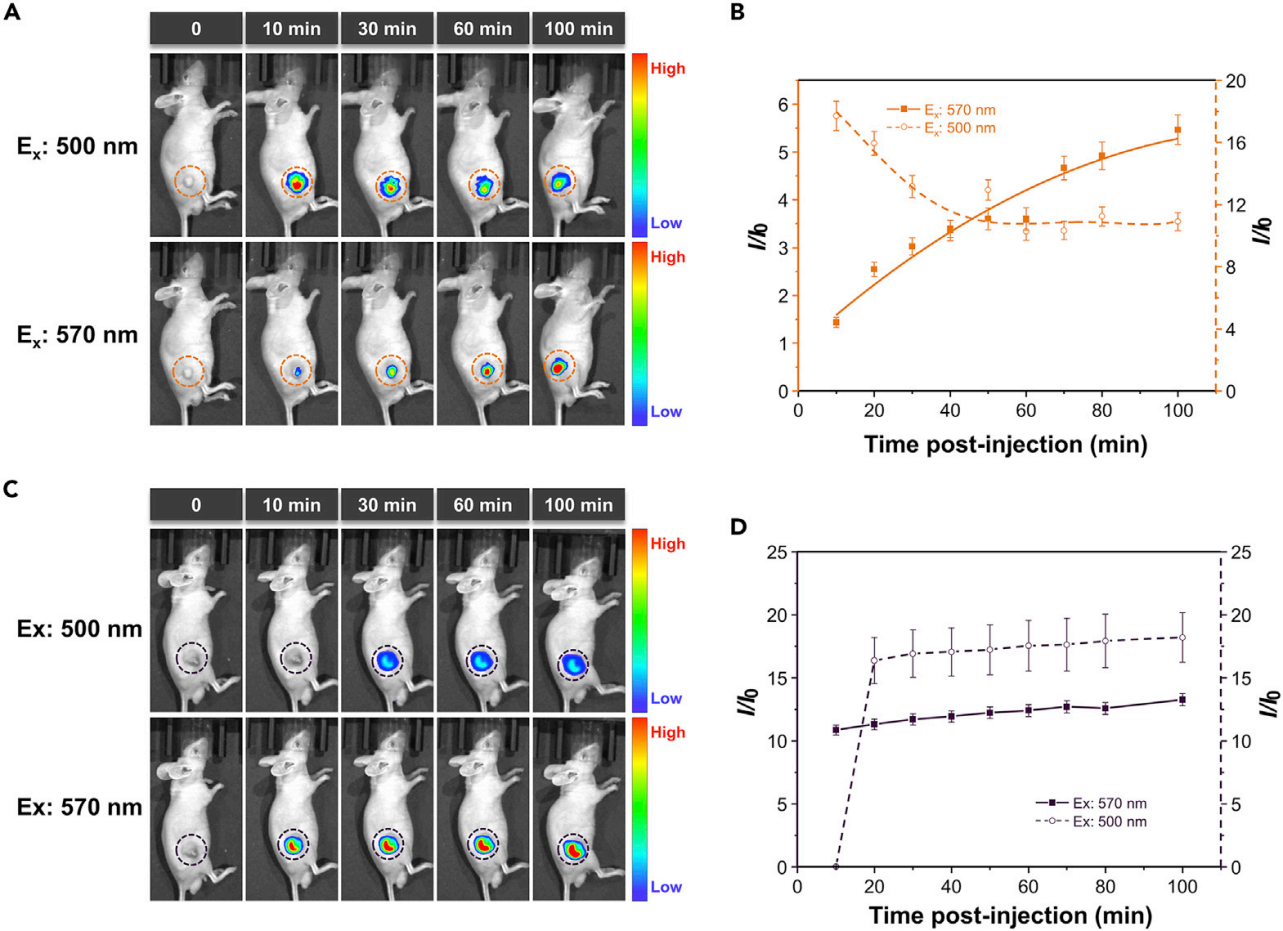
Hypoxia-activated NIR fluorescence imaging of TBTO NPs *in vivo* (A and C) Time-lapse NIR fluorescence imaging of mice before (time point: 0 min) and after intratumoral injection with (A) TBTO NPs and (C) TBT NPs (50 μL, 1 mg/mL), respectively. The fluorescence signals were collected upon excitation with 500 nm and 570 nm, respectively. (B and D) Semiquantitative analysis of fluorescence intensities in the tumor site of the mouse injected with (B) TBTO NPs and (D) TBT NPs as a function of time. Data are represented as mean ± SD from three replicates.

Encouraged by the *in vitro* hypoxia-responsibility of TBTO NPs, we subsequently investigated hypoxia-activated NIR imaging in tumor-bearing mice. Tumor allografts were constructed via subcutaneous implantation of HeLa cells into BALB/c mice. To monitor the conversion of TBTO to TBT in real-time, two mice were administered TBTO NPs (Figure 4A) and TBT NPs (Figure 4C) via intratumoral injection, respectively. Both mice were transferred to the small animal imaging system and imaged with excitation filters of 500 nm and 570 nm and corresponding NIR emission filters of 720 and 740 nm after different time intervals post-injection. As illustrated in Figures 4A and 4B, for the mouse injected with TBTO NPs, the fluorescence signal collected with the excitation filter of 500 nm gradually decreased, whereas the signal from 570 nm excitation gradually increased, demonstrating that TBTO underwent a reduction reaction in the hypoxic tumor. TBT NPs injected mouse, on the other hand, exhibited almost constant NIR fluorescence intensities within 100 min post-injection upon excitation with either 500 nm or 570 nm (Figures 4C and 4D), because TBT is inactive to the tumorous microenvironment. Since a shorter wavelength impedes the penetration in deep tissue, using a longer excitation wavelength never goes wrong for *in vivo* NIR imaging. The transition process was further reproduced using more mice (Figure S18). Moreover, the good photostability of TBTO NPs and TBT NPs was confirmed using the small animal imaging system (Figure S19). Thus, we could conclude that TBTO is promising as a hypoxia-activated NIR imaging agent. We next examined the tumor-targeting specificity and persistence of TBTO NPs via tail intravenous injection. As shown in Figures S20A and S20B, at 6 h post-injection, an obvious NIR fluorescence signal upon 570 nm excitation was detected in tumor and the intensity persisted more than 24 h. *Ex vivo* images of major organs and tumors at the time point of 24 h post-injection further verified the remarkable accumulation of TBTO NPs in tumors (Figure S20C). *Ex vivo* colocalization of TBTO signal in tumor site with a commercial hypoxia imaging kit via an immunofluorescence staining method was illustrated in Figure S21 (see supplemental information for experimental details).

### PA imaging of TBTO NPs *in vivo*

**Figure 5:**
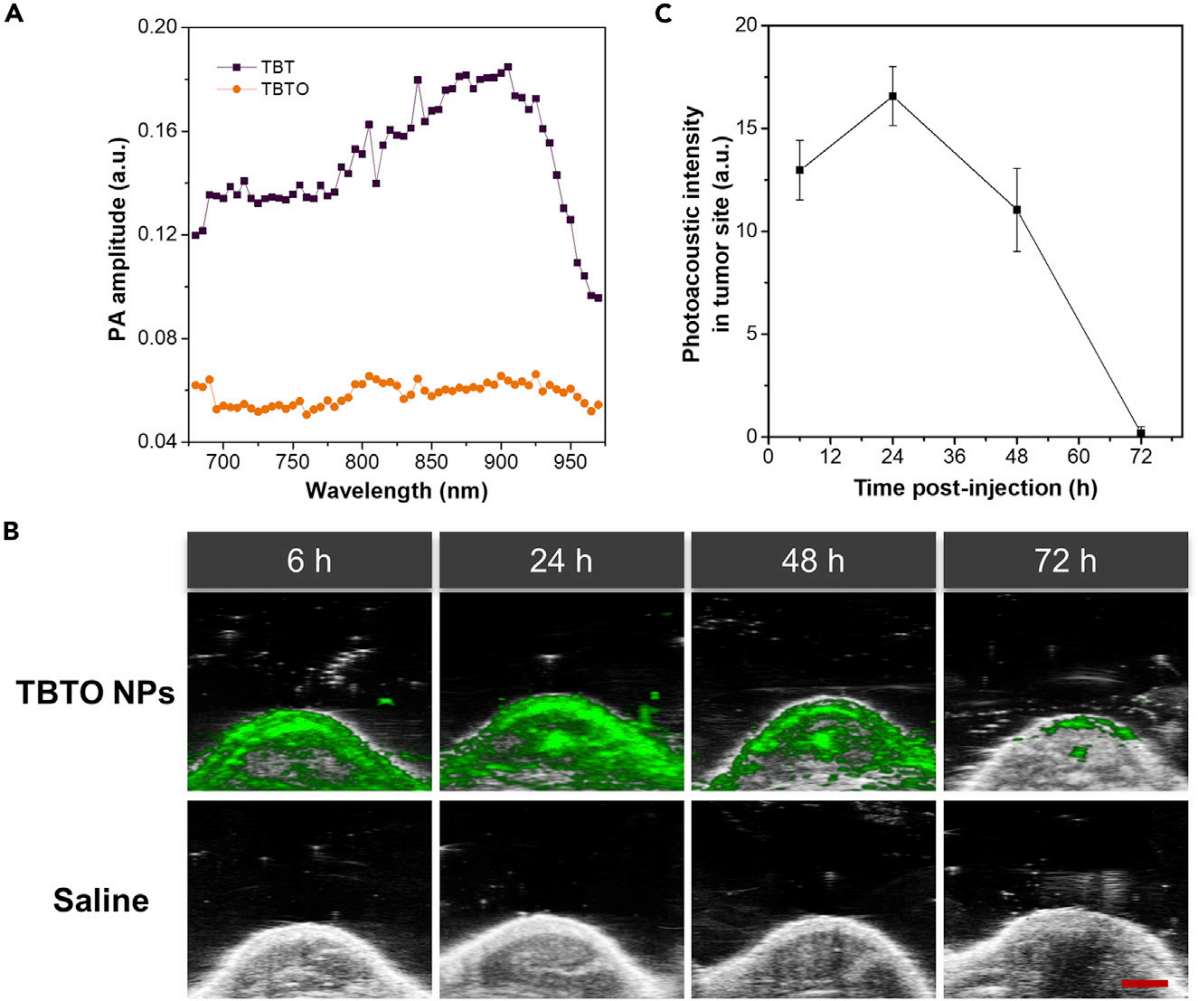
Hypoxia-activated PA Imaging of TBTO NPs (A) *In vitro* PA spectra of TBTO and TBT. PA amplitude corresponding to each compound was plotted as a function of wavelength. (B) *In vivo* PA images of the tumor site in the mice after tail intravenous injection with TBTO NPs (200 μL, 1 mg/mL) or saline (200 μL) as control. Scale bar represents 2 mm. (C) Semiquantitative analysis of average PA intensities in the tumor site of the mice injected with TBTO NPs as a function of time. Data are represented as mean ± SD from three replicates.

In the primary test, PA signals of both TBTO and TBT solutions were tested. As illustrated in Figure 5A, TBT showed a fairly strong PA signal whereas almost no PA signal was detected from TBTO under the same experimental conditions. We also performed *in vitro* PA imaging of TBTO NPs and TBT NPs aqueous solution (1 mg/mL) in fluorinated ethylene propylene tubes which were inserted into the tissue-mimicking phantom (Figure S22). In addition, PA signal of TBT could be detected *in vivo* upon intravenous administration of TBT NPs in tumor-bearing mice (Figure S23). These results suggested that TBTO can be potentially utilized as a hypoxia-triggered PA imaging agent with turn-on characteristics. To verify that, TBTO NPs were administered to tumor-bearing mice intravenously via tail vein injection, and *in vivo* PA images were acquired at 6 h, 1 d, 2 d, and 3 d following injection (Figure 5B). Semiquantitative analysis of average PA intensities in the tumor site at different time points was also performed (Figure 5C). It was observed that a strong PA signal was detected after 6 h post-injection resulting from the responsiveness of TBTO to hypoxia. Even after 3 days, the PA signal could still be detected, suggesting the utility of TBTO NPs for long-term tracking of the tumor.

Lastly, we evaluated the physiological clearance of TBTO NPs from mice, which is highly related to the biosafety for *in vivo* applications. As depicted in Figure S24A, upon intravenous injection of TBTO NPs via the tail vein, TBTO NPs could be eliminated from all major organs within 72 h. In the case of intratumoral injection, there were no PA signals detected in major organs after 48 h, and the PA signal in the tumor site was clearly presented with high contrast and then slowly cleared out until 72 h (Figure S24B). H&E staining results of the major organs (heart, liver, spleen, lung, and kidney) indicated the good biosafety and biocompatibility of TBT NPs and TBTO NPs (Figure S25). We could conclude from all these results that TBTO NPs is significantly promising for long-term tumor imaging as a hypoxia-activated PA probe with appropriate biocompatibility.

## Discussion

In summary, we rationally designed a hypoxia-activable probe TBTO, which is capable of being converted to AIE-active TBT in a hypoxic microenvironment. TBTO could be facilely synthesized in a few steps with a high yield. The typical D-A-D structure of TBT led to a strong TICT effect, which red-shifted the fluorescence emission to the NIR region and promoted the generation of PA signal. The fluorescence emission of TBT exhibited a positive solvatochromic effect with a bathochromic shift and quenched emission along with the increasing of solvent polarity, indicating a typical TICT phenomenon. The conversion of TBTO to TBT in a reductive environment was confirmed by fluorescence spectra and ^1^H-NMR. *In vitro* responsiveness of TBTO in hypoxic cells was confirmed by monitoring the fluorescence quenching of TBTO signal. *In vivo* assessments revealed that TBTO with good biocompatibility was highly responsive to the hypoxic tumor and well-performed in NIR fluorescence and PA dual-mode tumor imaging. This work presents a promising method for clinical imaging taking advantage of the specific microenvironment in a diseased region, which might be useful in tumor diagnostics, imaging-guided surgical interventions, and treatment efficacy evaluations.

### Limitations of the study

We have presented a hypoxia-responsive probe TBTO allowing for NIR-I imaging and more importantly PA imaging in solid tumors. Although the fluorescence emission could cover the NIR-I area, NIR-II fluorophores featuring longer absorption and emission wavelengths are worth investigating in the future for hypoxia imaging in deep tissue with high resolution (Li et al., 2020b; Sun et al., 2019; Tu et al., 2019; Xu et al., 2020c).

### Resource availability

#### Lead contact

Further information and requests for reagents and resources should be directed to and will be fulfilled by the lead contact, Ben Zhong Tang (tangbenz@ust.hk).

#### Materials availability

All unique reagents generated in this study are available from the lead contact with a completed Materials Transfer Agreement.

#### Data and code availability

The published article includes all datasets generated or analyzed during the study.

## Methods

All methods can be found in the accompanying transparent methods supplemental file.

## Supplemental information

Document S1. Transparent methods, Figures S1–S25, Table S1, and Scheme S1 and S2

## Footnotes

Supplemental information can be found online at https://doi.org/10.1016/j.isci.2021.102261.
