# Supplementary material for: Hypoxia-activated probe for NIR fluorescence and photoacoustic dual-mode tumor imaging
Source: iScience. 2021 Mar 2;24(3):102261. doi: 10.1016/j.isci.2021.102261 (PMC7973868; doi:10.1016/j.isci.2021.102261)
Supplement: Document S1. Transparent methods, Figures S1–S25, Table S1, and Scheme S1 and S2 [file mmc1.pdf]

iScience, Volume *24*

Supplemental information

Hypoxia-activated probe for NIR fluorescence and photoacoustic dual-mode tumor imaging

Meng Li, Huan Li, Qian Wu, Niu Niu, Jiachang Huang, Lingmin Zhang, Ying Li, Dong Wang, and Ben Zhong Tang

Supplemental Data

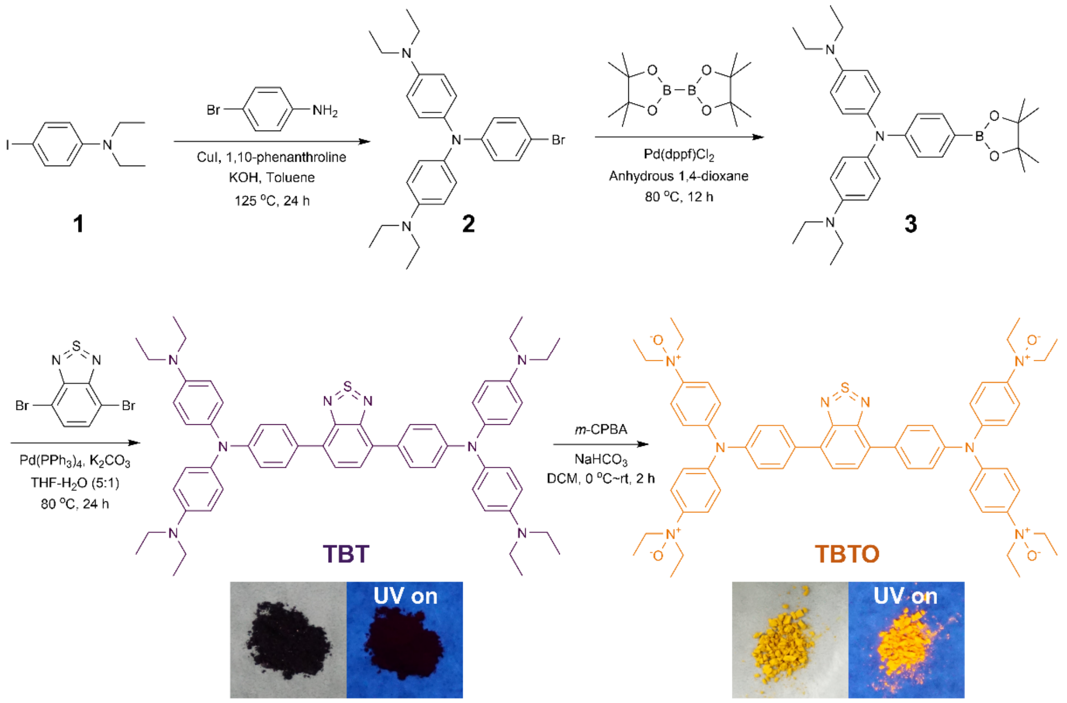

Scheme S1. Synthetic Route of TBT and TBTO, Related to Scheme 1.

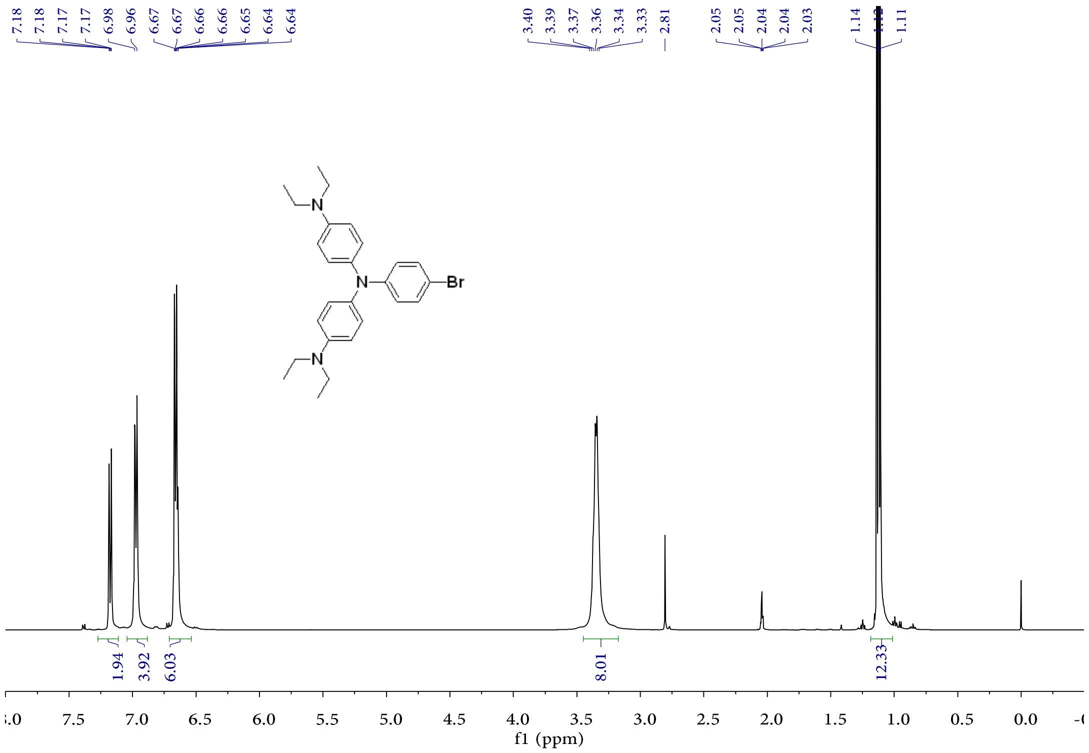

Figure S1. ^{1}H-NMR of Compound 2 in d_6-Acetone, Related to Scheme 1.

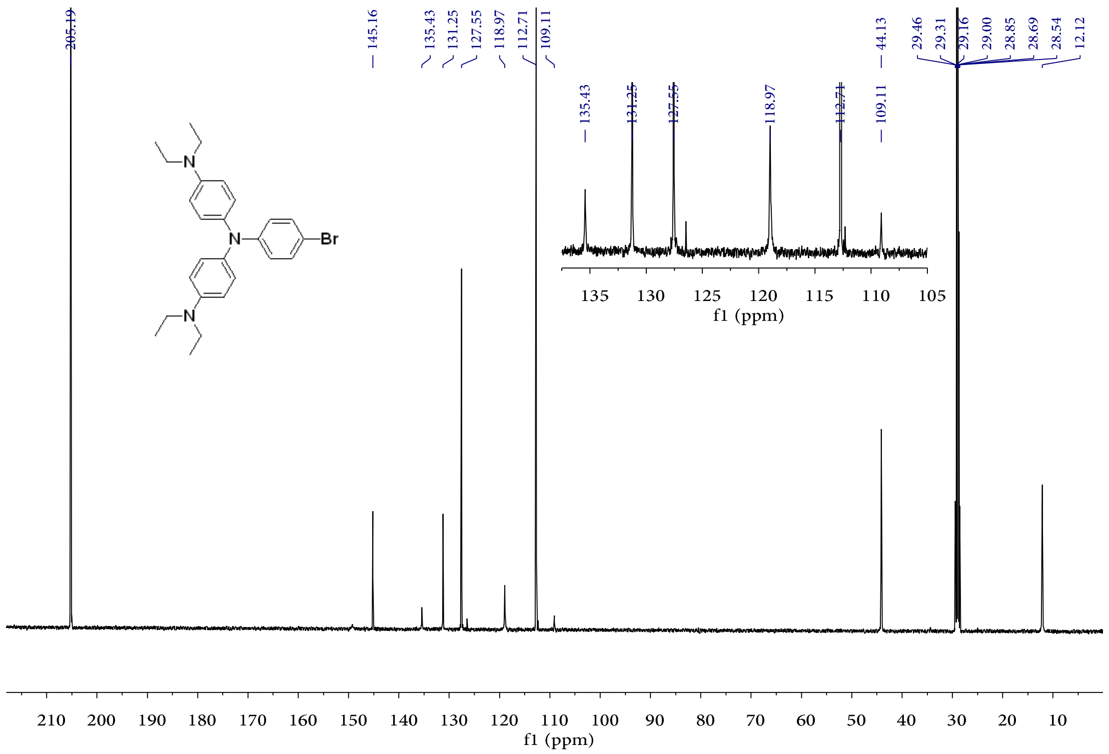

Figure S2. ^{13}C-NMR of Compound 2 in d_6-Acetone, Related to Scheme 1.

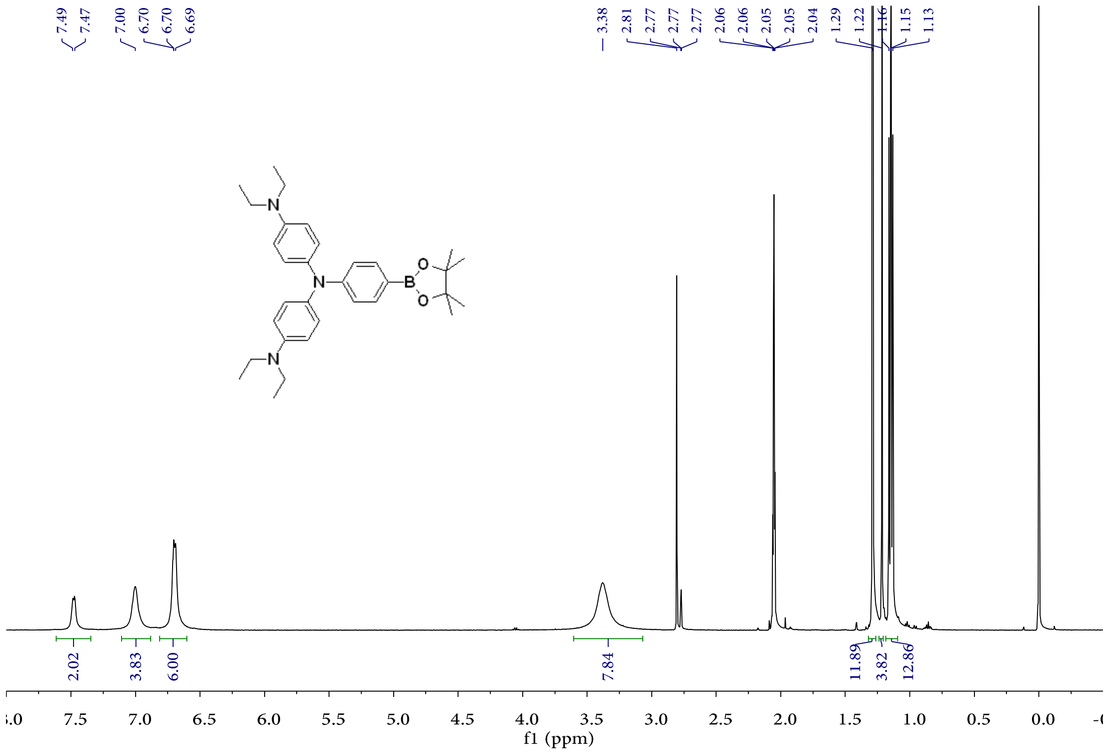

Figure S3. ^{1}H-NMR of Compound 3 in d_6-Acetone, Related to Scheme 1.

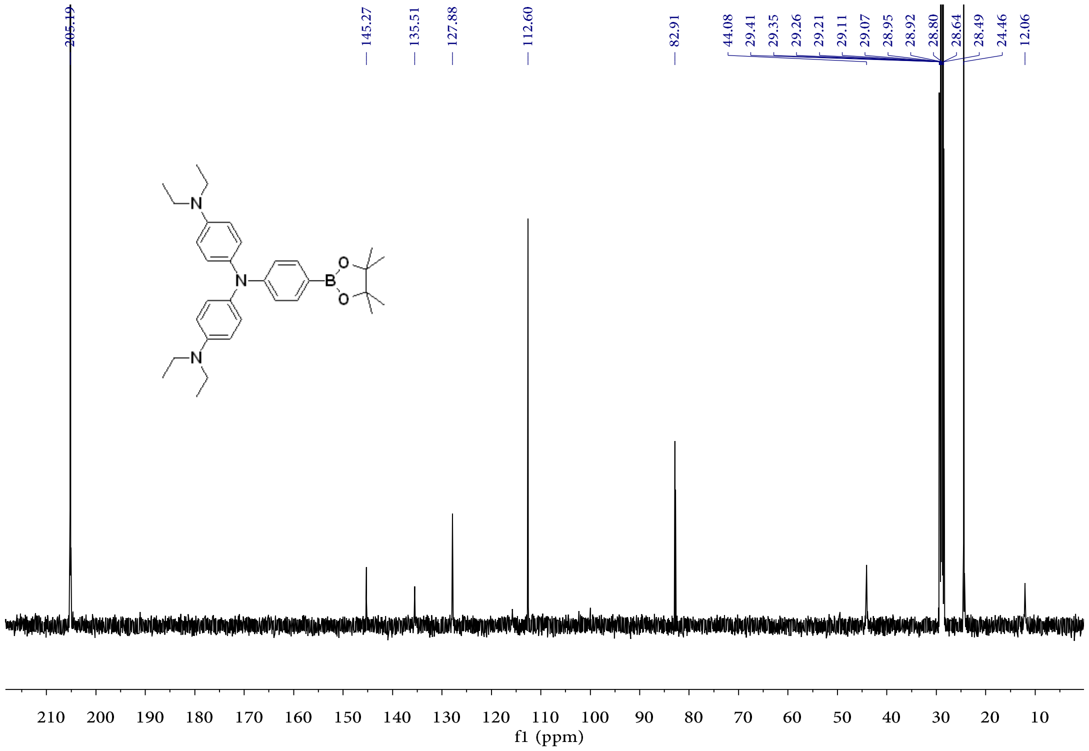

Figure S4. ^{13}C-NMR of Compound 3 in d_6-Acetone, Related to Scheme 1.

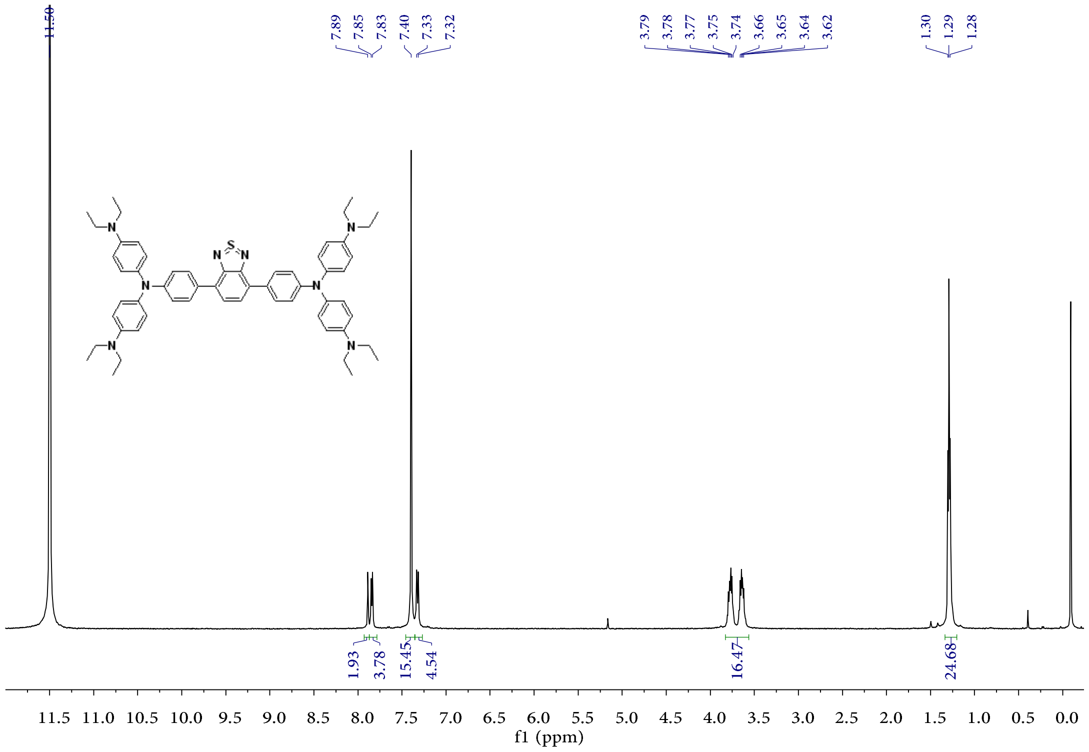

Figure S5. ^{1}H-NMR of TBT in *d*-Trifluoroacetic Acid, Related to Scheme 1.

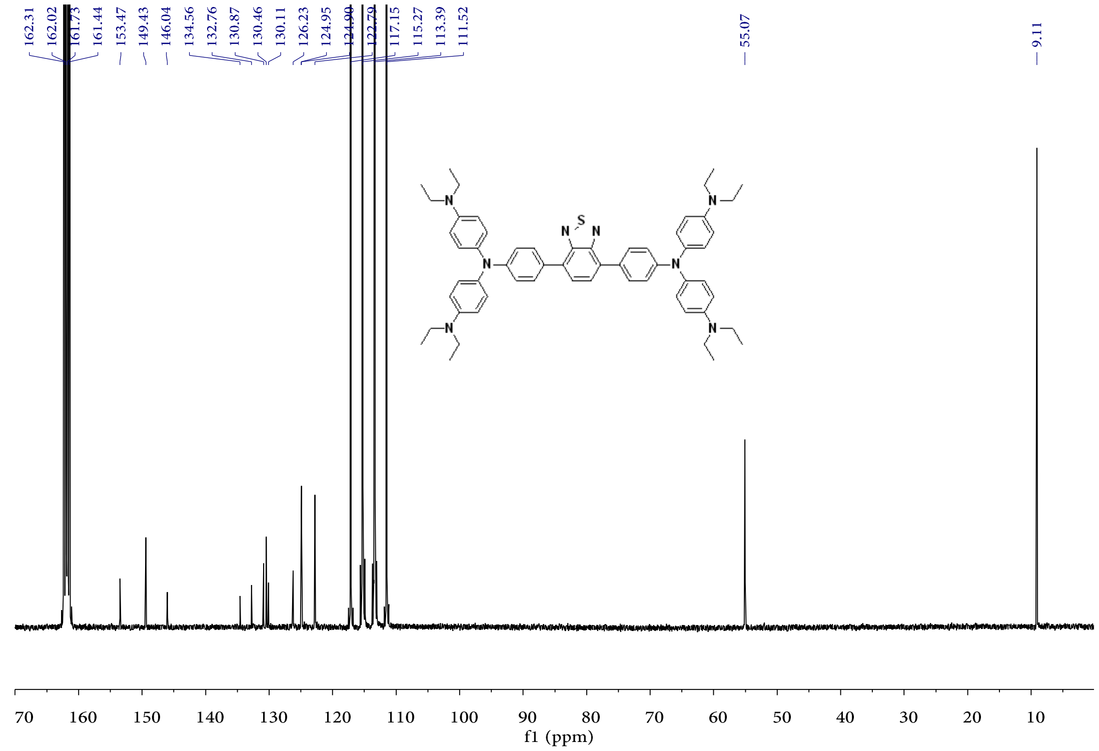

Figure S6. ^{13}C-NMR of TBT in *d*-Trifluoroacetic Acid, Related to Scheme 1.

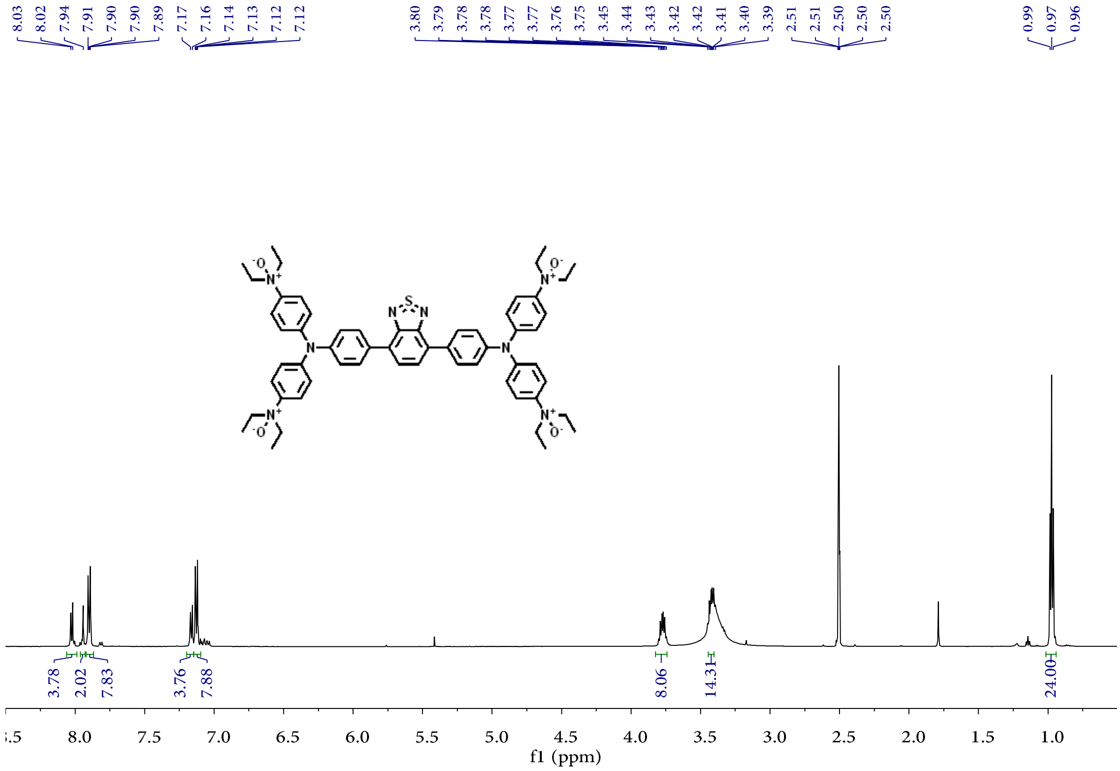

Figure S7. ^{1}H-NMR of TBTO in d_6-Dimethyl Sulfoxide, Related to Scheme 1.

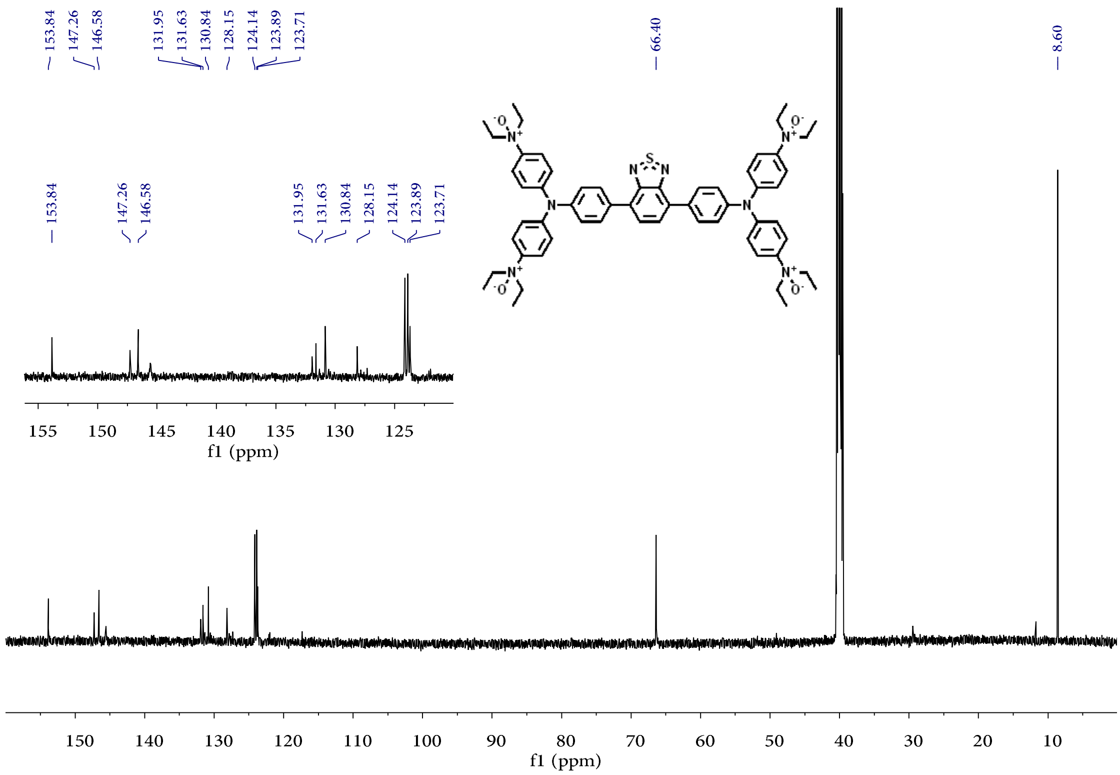

Figure S8. ^{13}**C-NMR of TBTO in** d^6**-Dimethyl Sulfoxide, Related to Scheme 1.**

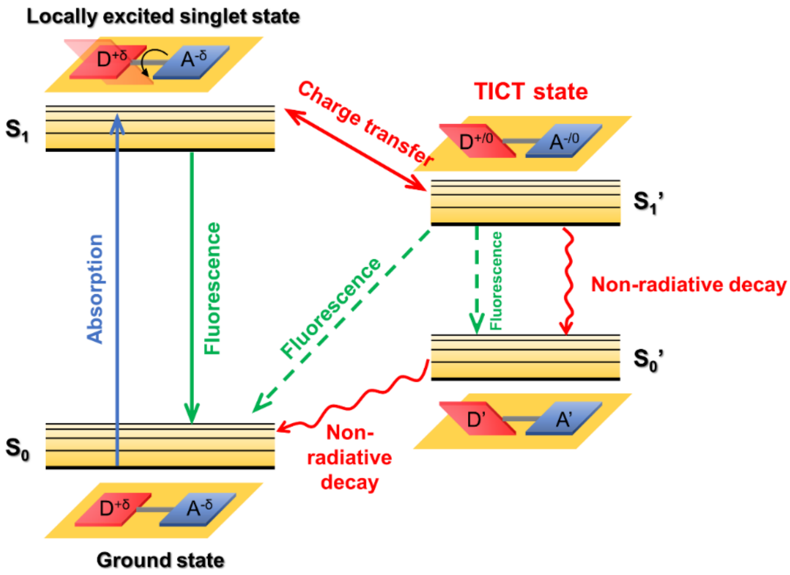

Scheme S2. Jablonski Diagram of TICT Dynamics Referred to Literature Reported (Liu et al., 2019; Sasaki et al., 2016), Related to Scheme 1.

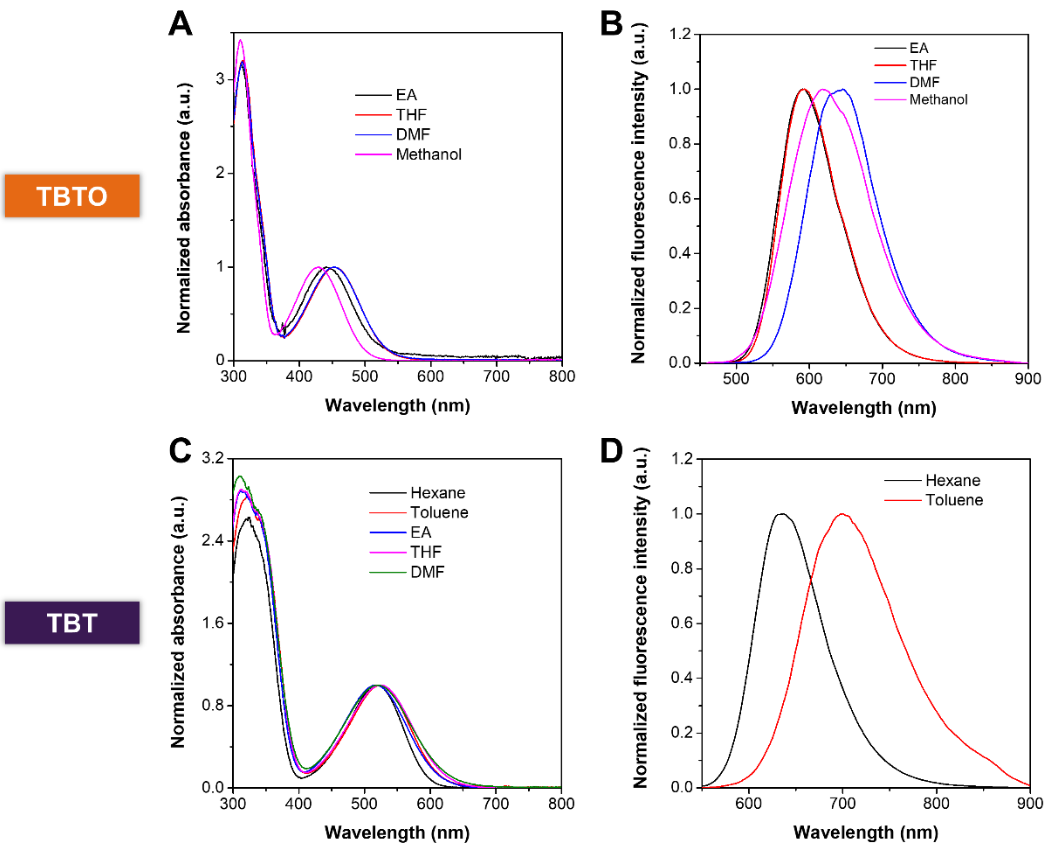

Figure S9. Normalized Absorption and Emission Spectra of TBT and TBTO in Different Solvents, Related to Figure 2. [TBTO], [TBT] = 20 μM.

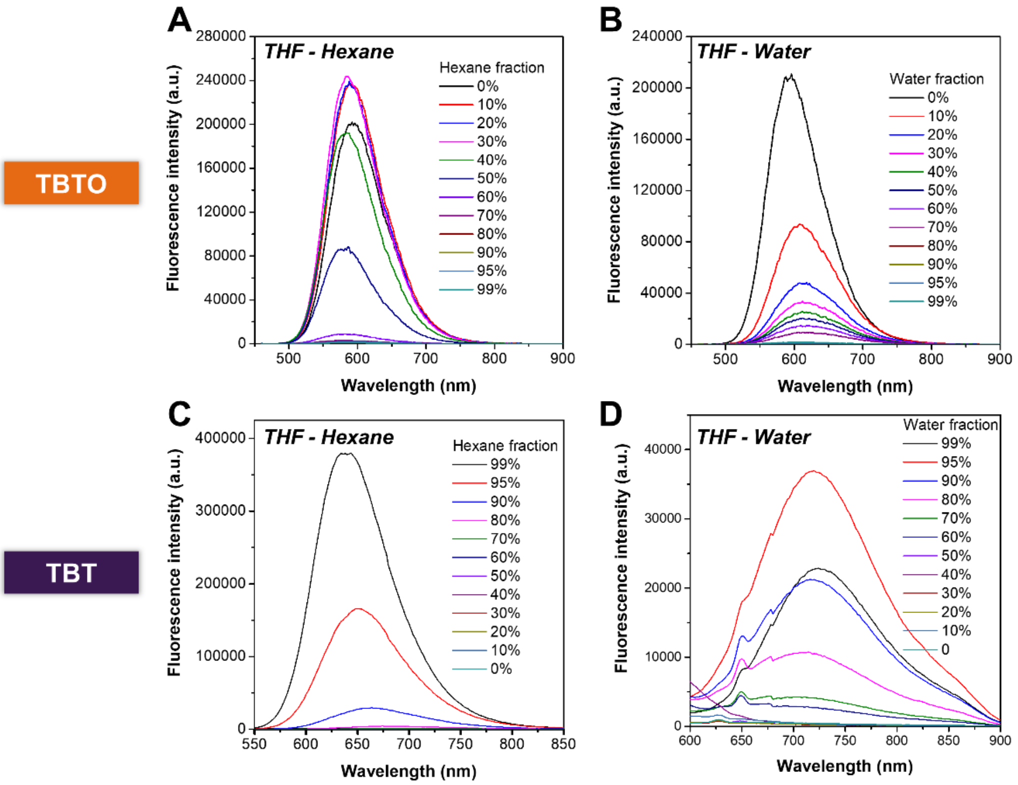

Figure S10. Solvatochromic Effect of TBT and TBTO in the Mixed Solvent System, Related to Figure 2.

Fluorescence emission spectra of (A) TBTO and (C) TBT in THF with different fractions of hexane. Fluorescence emission spectra of (B) TBTO and (D) TBT in THF with different fractions of water. [TBTO], [TBT] = 20 μM.

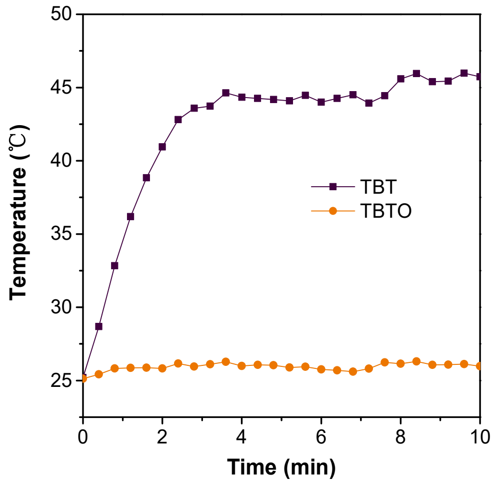

Figure S11. Photothermal Conversion Effect of TBTO and TBT in DMF upon Irradiation with a 660 nm Laser, Related to Scheme 1.

[TBT], [TBTO] = 500 μM. Laser power: 0.5 W/cm^2.

Table S1. Characterizations of TBT NPs and TBTO NPs in Water, Related to Figure 3.

Materials	Size	Zeta potential	$\lambda_{abs, max}$	$\lambda_{em, max}$	QY
TBTO NPs	50.3 ± 0.7 nm	-22.8 ± 3.8 mV	441 nm	596 nm	3.2%
TBT NPs	103.0 ± 0.6 nm	-41.2 ± 1.2 mV	542 nm	724 nm	0.5%

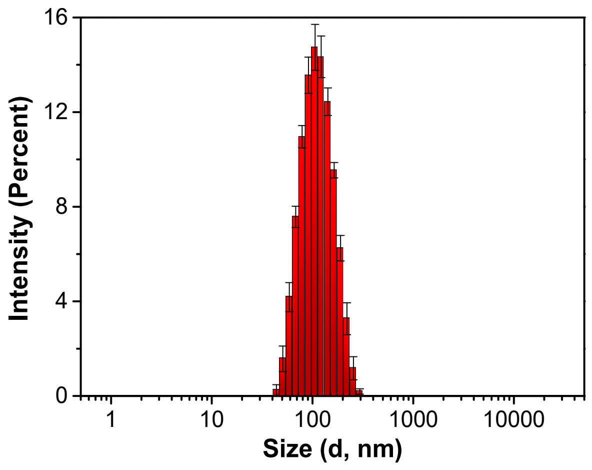

Figure S12. The Size Distribution of TBT NPs in Water, Related to Figure 3.

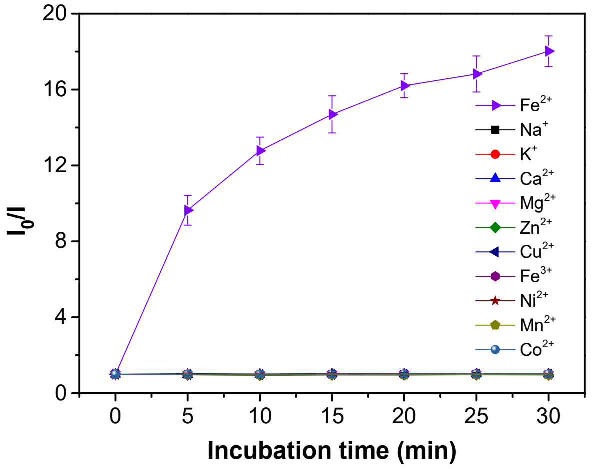

Figure S13. In Vitro Responsiveness of TBTO NPs to Different Metal Ions, Related to Figure 3.

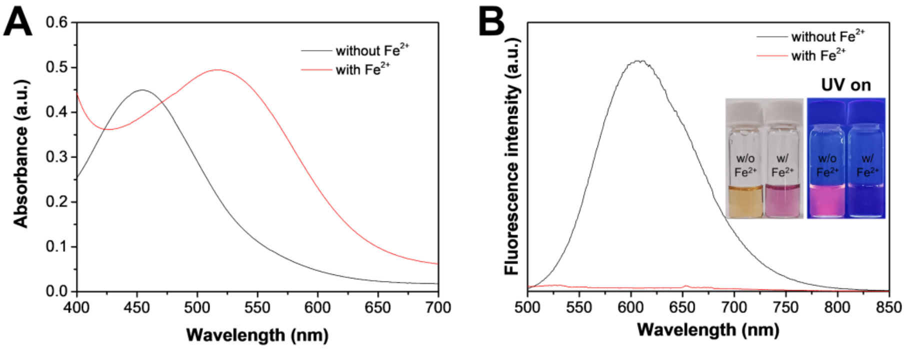

Figure S14. In Vitro Responsiveness of TBTO NPs to Fe(II), Related to Figure 3.

(A) The absorption spectra of TBTO NPs after incubation with or without Fe(II) at 37°C for 2 h. (B) The fluorescence emission spectra of TBTO NPs after incubation with or without Fe(II) at 37°C for 2 h (Insert: the pictures of TBTO NPs solution with or without Fe(II) incubation taken under ambient light or UV lamp).

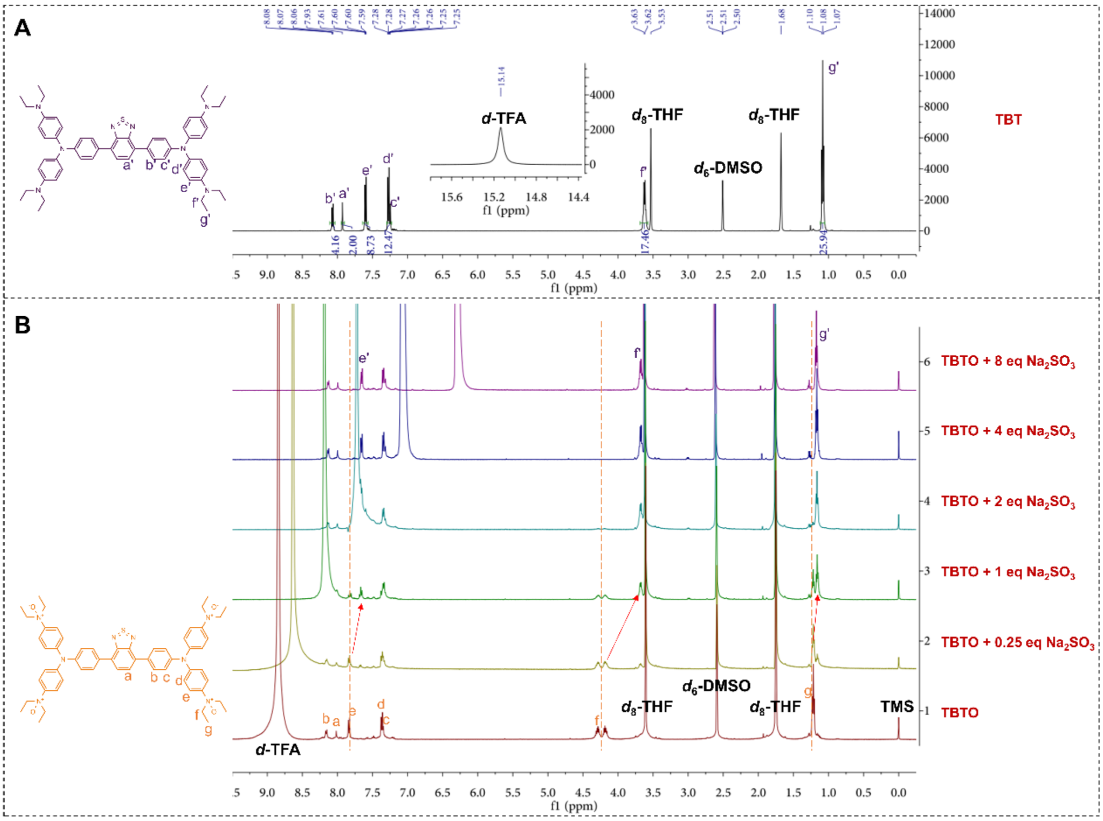

Figure S15. In Vitro Transition of TBTO to TBT Verified by ^{1}H-NMR, Related to Figure 3.
(A) ^{1}H-NMR of TBT in 800 μL of deuterated THF-DMSO-TFA (3.5:3.5:1).
(B) To the solution of TBTO (2 μmol) in 800 μL of deuterated THF-DMSO-TFA (3.5:3.5:1), was added Na_2SO_3 in D_2O (1 M): 2 μL (0.25 eq), 8 μL (1 eq), 16 μL (2 eq), 32 μL (4 eq), and 64 μL (8 eq).

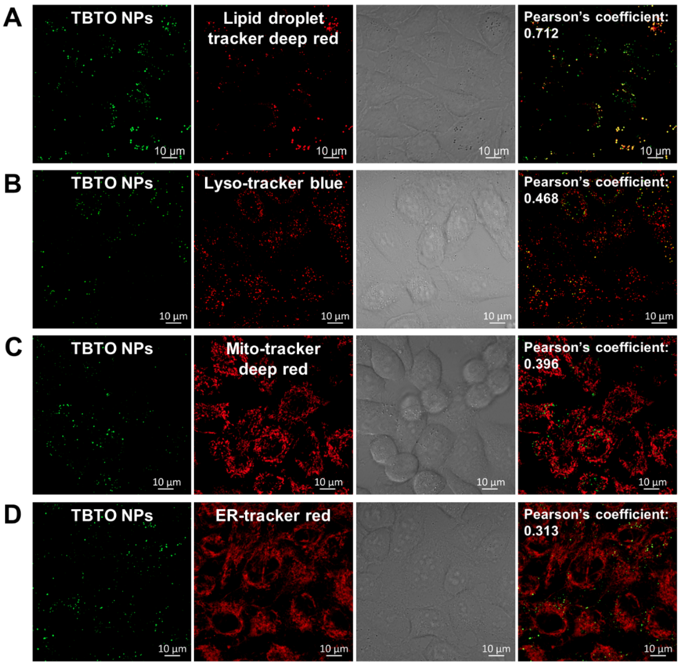

Figure S16. Colocalization Images of TBTO NPs with Different Organelle Trackers,

Related to Figure 3.

CLSM images of HeLa cells incubated with 50 μM TBTO NPs (λ_{ex}: 488 nm; λ_{em}: 610-700 nm) and then 1 μM different organelle trackers: (A) lipid droplet tracker deep red (λ_{ex}: 640 nm; λ_{em}: 645-700 nm), (B) lyso-tracker blue (λ_{ex}: 405 nm; λ_{em}: ＜505 nm), (C) mito-tracker deep red (λ_{ex}: 640 nm; λ_{em}: 645-700 nm), and (D) ER-tracker red (λ_{ex}: 561 nm; λ_{em}: ＜610 nm).

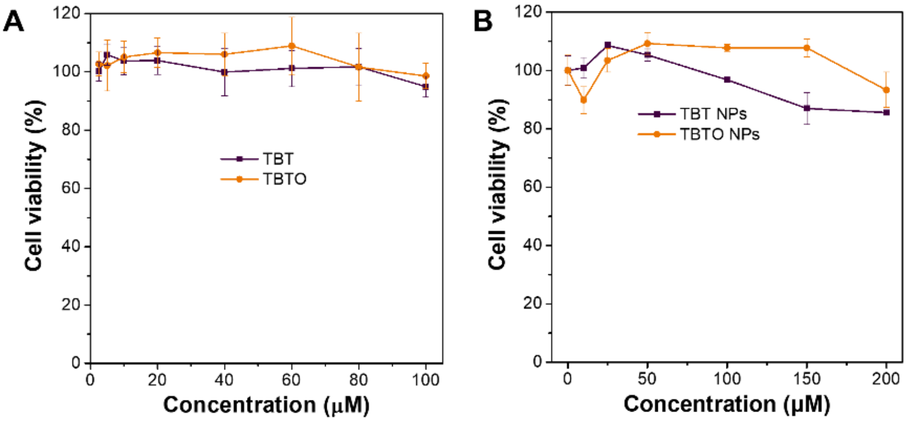

Figure S17. In Vitro Cell Viability of HeLa Cells Against TBT and TBTO in the Form of (A) Bare Molecules or (B) Nanoparticles after Incubation for 24 h, Related to Figure 3.

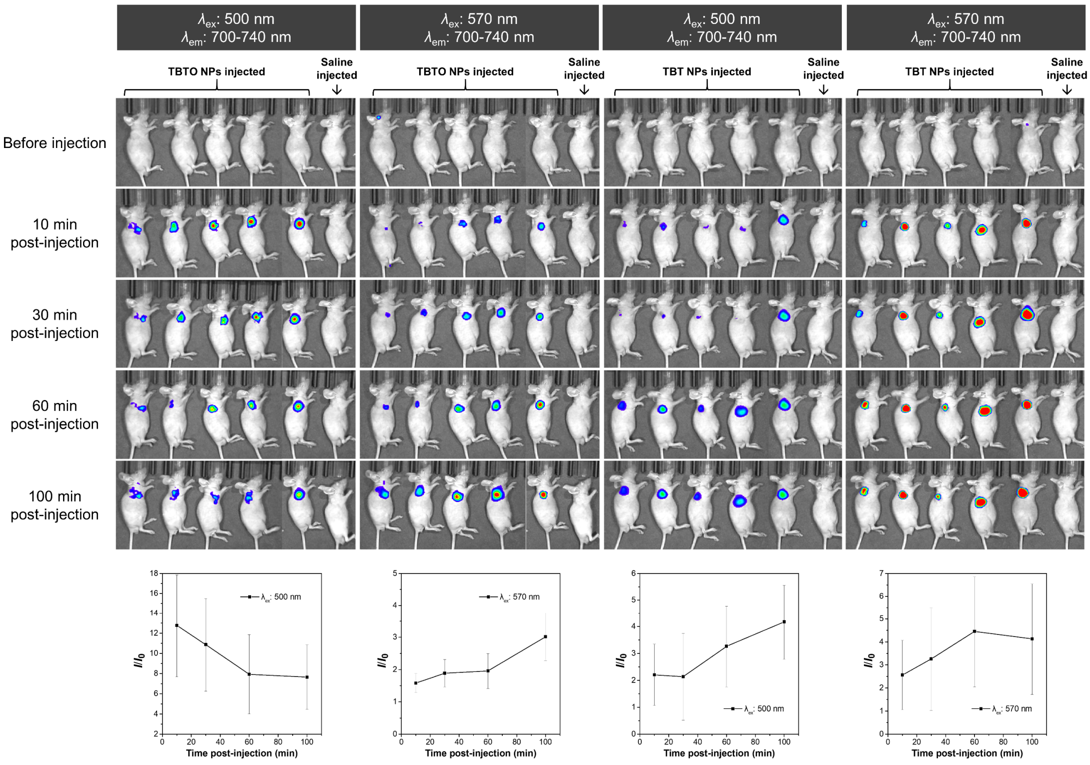

Figure S18. Hypoxia-activated Fluorescence Imaging of TBTO NPs in Vivo, Related to Figure 4.

Time-lapse NIR fluorescence imaging of mice before and after intratumoral injection with TBTO NPs or TBT NPs (50 μL, 1 mg/mL) and semiquantitative analysis of fluorescence intensities in the tumor site of the mice as a function of time. The fluorescence signals were

collected upon excitation with 500 nm and 570 nm, respectively.

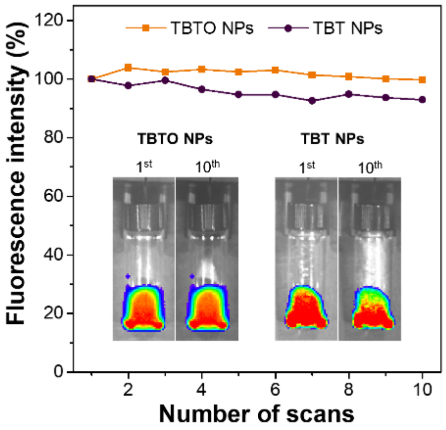

Figure S19. Photostability of TBTO NPs and TBT NPs, Related to Figure 4.

The fluorescence intensities of TBTO NPs (λ_{ex}: 500 nm) and TBT NPs (λ_{ex}: 570 nm) upon illumination with the laser in the small animal imaging system for 10 times.

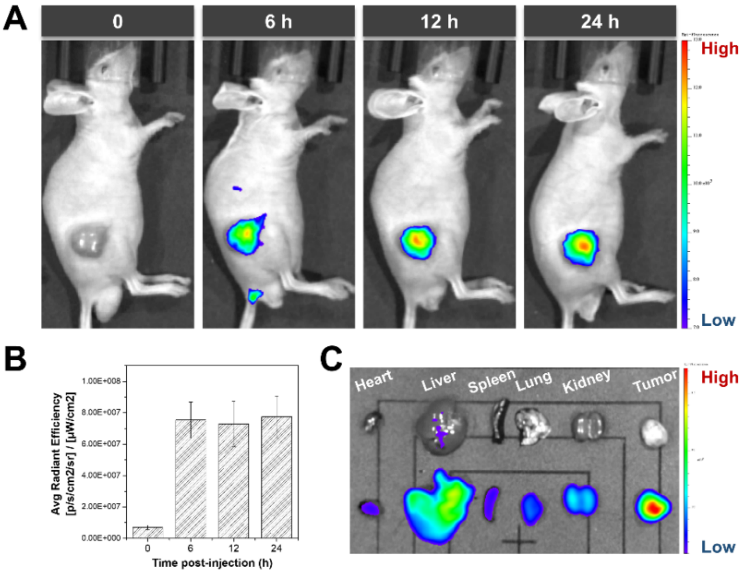

Figure S20. Fluorescence Imaging of TBTO NPs in Tumor-bearing Mice via Tail Intravenous Injection, Related to Figure 4.

(A) Time-lapse NIR fluorescence imaging of mice before (time point: 0 min) and after tail intravenous injection with TBTO NPs.

(B) Semiquantitative analysis of fluorescence intensities in the tumor site of the mouse injected with TBTO NPs.

(C) Ex vivo NIR fluorescence images of major organs and tumors after injection with PBS (the upper row) and TBTO NPs (the lower row) for 24 h.

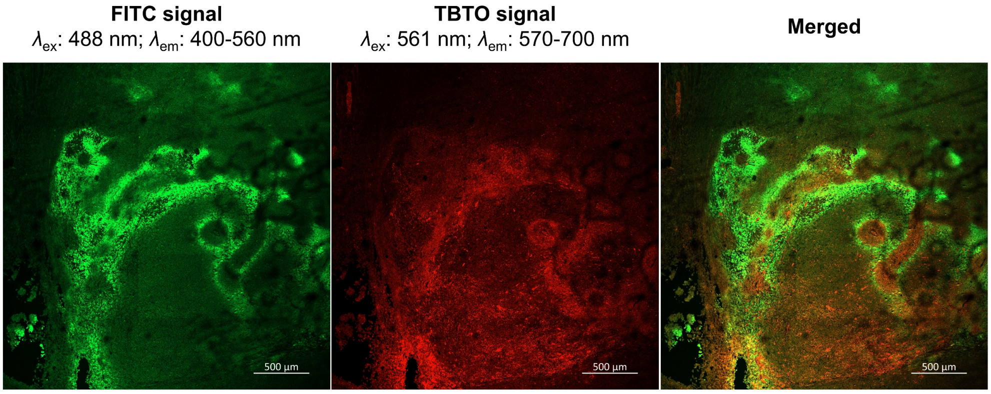

Figure S21. Ex Vivo Colocalization of TBTO Signal in Tumor Site with a Commercial Hypoxia Imaging Kit Using Immunofluorescence Staining Method, Related to Figure 4.

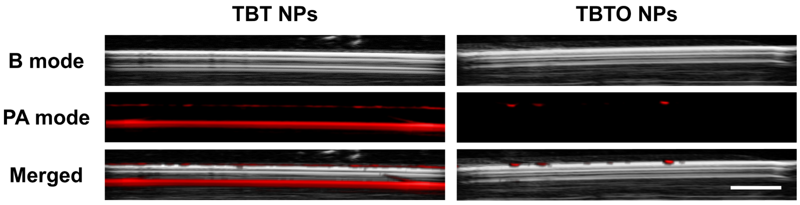

Figure S22. In Vitro PA Imaging of TBTO NPs and TBT NPs in FEP Tubes, Related to Figure 5. Scale bar represents 2 mm.

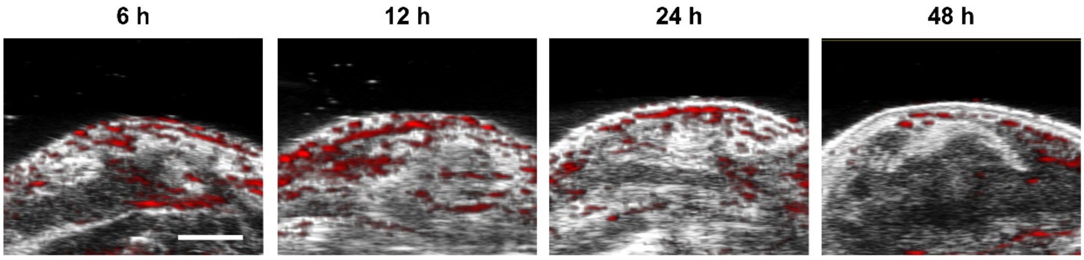

Figure S23. In Vivo PA Imaging with TBT NPs, Related to Figure 5.

In vivo PA images of the tumor site in the mice after tail intravenous injection with TBT NPs (200 μL, 1 mg/mL). Scale bar represents 2 mm.

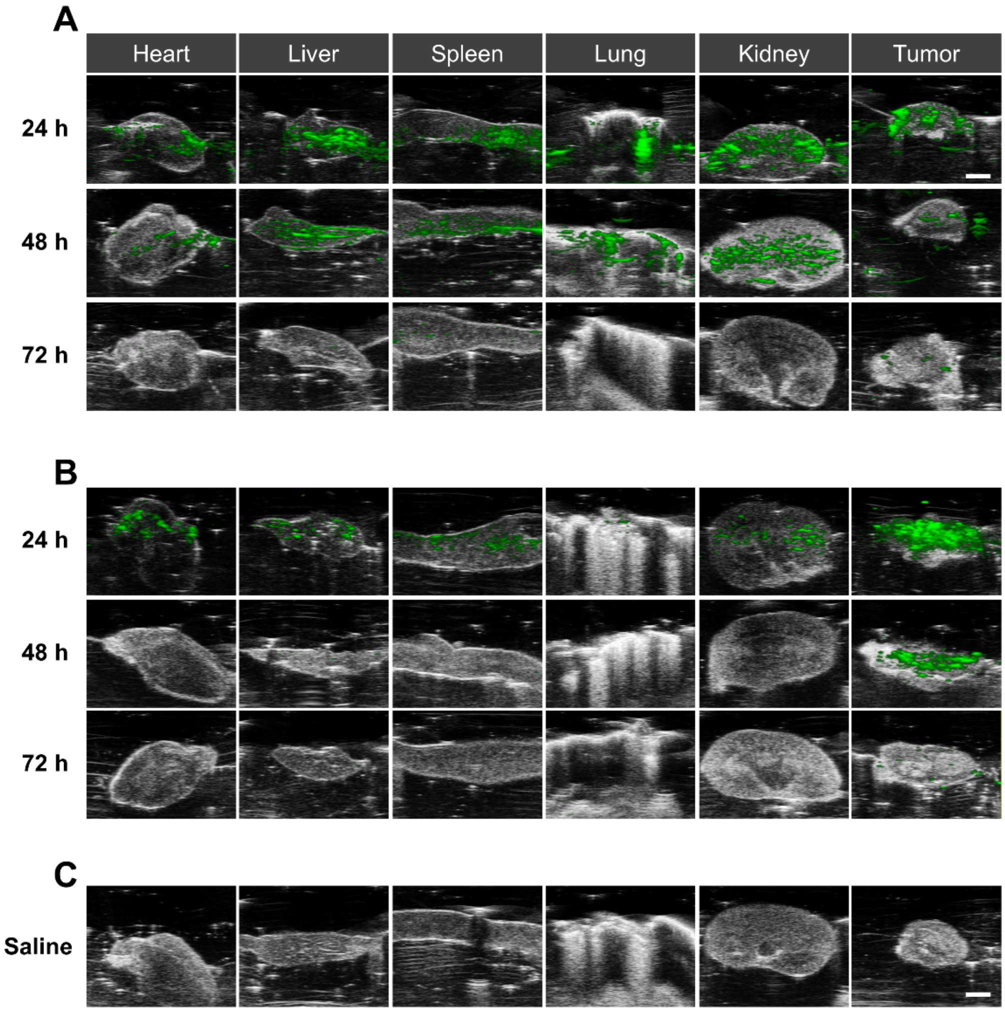

Figure S24. Bioelimination of TBTO NPs from Major Organs and Tumors, Related to Figure 5.

(A) PA images of major organs and tumors at different time points after tail intravenous injection of TBTO NPs.

(B) PA images of major organs and tumors at different time points after intratumoral injection of TBTO NPs.

(C) PA images of major organs and tumors at different time points after tail intravenous injection of saline. Scale bar: 2 mm.

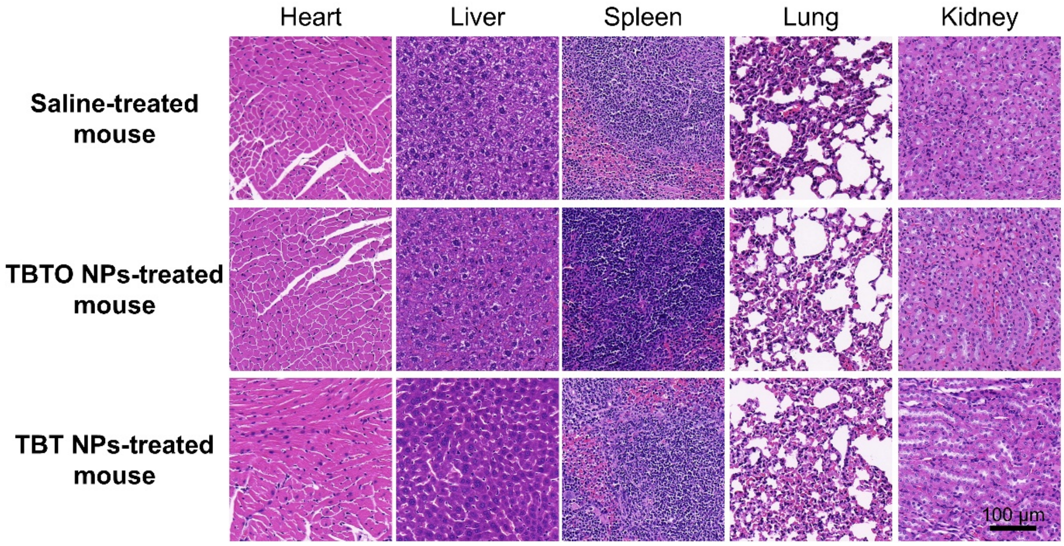

Figure S25. H&E Staining Images of the Major Organs after Intravenous Injection of (A) TBTO NPs and (B) TBT NPs via Tail Vein for 72 h, Related to Figure 5.

Transparent Methods

Materials and instruments:

DSPE-mPEG2000 with a methoxy terminal group (1,2-distearoyl-sn-glycero-3-phosphoethanolamine-N-[methoxy(polyethylene glycol)-2000], CAS: 147867-65-0) was purchased from Xi'an Ruixi Biological Technology Co., Ltd. ER-Tracker Red (Cat. No. C1041) for endoplasmic reticulum staining was purchased from Beyotime Biotechnology (Shanghai, China). LysoBlue (Cat. No. KGMP006-1) for lysosome staining was purchased from Jiangsu KeyGEN BioTECH Co., Ltd (Nanjing, China). HCS LipidTOX Deep Red Neutral (Cat. No. H34477) for lipid droplet staining and MitoTracker Deep Red FM (Cat. No. M22426) for mitochondria staining were purchased from Thermo Fisher Scientific Inc (USA). Dulbecco's Modified Eagle Medium (DMEM) with high glucose, phosphate buffer saline, fetal bovine serum (FBS), and penicillin/streptomycin were purchased from Thermo Fisher Scientific Inc. Hypoxyprobe Green Kit-1 (Cat. No. HP6-100kit), a commercial hypoxia imaging kit containing pimonidazole HCl (Hypoxyprobe-1) and FITC-conjugated anti-pimonidazole mouse IgG_1 monoclonal antibody (FITC-MAb1), was purchased from Hypoxyprobe Inc (USA). All the chemicals and reagents were purchased from Shanghai Aladdin Biochemical Technology Co., Ltd. or J&K Scientific Ltd. and they were used as purchased without further purification. AnaeroPack™ anaerobic gas generator, 2.5 L rectangular sealed container, and anaero-indicators were purchased from Mitsubishi Chemical Corporation.

All the NMR spectra were recorded on a Bruker AVANCE III 500 MHz and 600 MHz (Bruker, Germany). High-resolution mass spectra (HR-MS) were recorded on an AB SCIEX Triple TOF 6600 (AB SCIEX, USA). UV-vis absorption spectra were recorded on a PerkinElmer Lambda 950 (Perkin-Elmer, USA). Fluorescence emission spectra were recorded on an FS 5 fluorescence spectrometer (Edinburgh Instruments, UK). The absolute photoluminescence quantum yield was determined by Hamamatsu Quantaurus-QY (Hamamatsu Photonics, Japan). The size distribution of nanoparticles was tested with a dynamic light scattering (DLS) method using a Malvern Zetasizer Nano series (Malvern Instruments, Inc., UK). Particle size and morphology were observed on a Hitachi HT7700 transmission electron microscope. CLSM images were taken on an LSM880 (Carl Zeiss AG, Germany). Photothermal conversion performance was monitored by an E6 IR-camera (FLIR, USA). In vivo fluorescence imaging was performed using an IVIS Spectrum live-animal imaging system (Perkin-Elmer, USA). PA imaging was performed using a Vevo LAZR-X (Fuji Film Visual Sonics, USA).

The synthesis of compound 2:

Compound 1 was synthesized according to known procedures and the ^{1}H-NMR datum matches that reported in the literature. (Tancini et al., 2012) To the solution of compound 1 (1.4 g, 5 mmol) and 4-bromoaniline (344 mg, 2 mmol) in 10 mL of toluene, was added KOH (1.8 g, 32 mmol), 1, 10-phenanthroline (36 mg, 0.2 mmol) and CuI (38 mg, 0.2 mmol). The resulting mixture was cooled to 0°C, degassed, and then flushed with N_2 for three cycles. The reaction was heated at 125°C under an N_2 atmosphere for 24 hours and then cooled down to room temperature. The reaction mixture was diluted in dichloromethane, washed with 1 M HCl

solution and brine, dried over anhydrous sodium sulfate, and concentrated in a vacuum. The crude mixture was purified by silica gel (200-300 mesh) column chromatography (petroleum ether: ethyl acetate = 25:1) to afford the pure product as an off-white solid (720 mg, 77% yield).
^{1}H NMR (500 MHz, Acetone-d_6) δ 7.27 – 7.11 (m, 2H), 6.97 (d, *J* = 8.6 Hz, 4H), 6.71 – 6.54 (m, 6H), 3.36 (p, *J* = 7.5 Hz, 8H), 1.12 (t, *J* = 7.1 Hz, 12H).
^{13}C NMR (126 MHz, Acetone-d_6) δ 145.16, 135.43, 131.25, 127.55, 118.97, 112.71, 109.11, 44.13, 12.12.
HR-MS (TOF) m/z: $[M+H]^+$ Calc'd for $C_{26}H_{33}N_3Br^+$ 466.1853 and 468.1832; Found 466.1850 and 468.1828.

The synthesis of compound 3:
To the solution of compound 2 (757 mg, 1.6 mmol) and bis(pinacolato)diboron (620 mg, 2.44 mmol) in 15 mL of 1.4-dioxane was added potassium acetate (798 mg, 8.14 mmol) and Pd(dppf)Cl_2 (50 mg). The resulting mixture was cooled to 0°C, degassed, and then flushed with N_2 for three cycles. The reaction was heated at 80°C under an N_2 atmosphere for 12 hours and then cooled down to room temperature. The reaction mixture was diluted in ethyl acetate, washed with brine three times, dried over anhydrous sodium sulfate, and concentrated in a vacuum. The crude mixture was purified by silica gel (200-300 mesh) column chromatography (petroleum ether: ethyl acetate = 10:1) to afford the pure product as a pale yellow solid (712 mg, 85% yield).
^{1}H NMR (500 MHz, Acetone-d_6) δ 7.48 (d, J = 8.1 Hz, 2H), 7.00 (s, 4H), 6.81 – 6.60 (m, 6H), 3.38 (m, 8H), 1.29 (s, 12H), 1.15 (t, J = 7.0 Hz, 12H).
^{13}C NMR (126 MHz, Acetone-d_6) δ 145.27, 135.51, 127.88, 112.60, 82.91, 44.08, 24.46, 12.06.
HR-MS (TOF) m/z: $[M+H]^+$ Calc'd for $C_{32}H_{45}BN_3O_2^+$ 514.3599; Found 514.3602.

The synthesis of TBT:
To the solution of compound 3 (712 mg, 1.4 mmol) and 4,7-dibromo-2,1,3-benzothiadiazole (136 mg, 0.46 mmol) in 10 mL of 1,4-dioxane and 2 mL of water, was added potassium carbonate (510 mg, 3.7 mmol) and 60 mg Pd(PPh_3)$_4$. The resulting mixture was cooled to 0°C, degassed, and then flushed with N_2 for three cycles. The reaction was heated at 100°C under an N_2 atmosphere for 24 hours and then quenched with water. The aqueous layer was extracted with dichloromethane three times, dried over sodium sulfate, and concentrated in a vacuum. The crude mixture was purified by silica gel (200-300 mesh) column chromatography (petroleum ether: ethyl acetate = 4:1) to afford the pure product as a magenta solid (403 mg, 89% yield).
^{1}H NMR (500 MHz, Trifluoroacetic acid-*d*) δ 7.89 (s, 2H), 7.84 (d, *J* = 8.1 Hz, 4H), 7.40 (s, 16H), 7.32 (d, *J* = 8.2 Hz, 4H), 3.71 (ddd, *J* = 66.6, 13.2, 7.2 Hz, 16H), 1.29 (t, *J* = 7.1 Hz, 24H).
^{13}C NMR (151 MHz, Trifluoroacetic acid-*d*) δ 153.47, 149.43, 146.04, 134.56, 132.76, 130.87, 130.46, 130.11, 126.23, 124.90, 122.79, 55.07, 9.11.
HR-MS (TOF) m/z: $[M+H]^+$ Calc'd for $C_{58}H_{67}N_8S^+$ 907.5204; Found 907.5226. $[M+2H]^{2+}$ Calc'd for $C_{58}H_{68}N_8S^{2+}$ 454.2638; Found 454.2653.

The synthesis of TBTO:

To the solution of TBT (90.7 mg, 0.1 mmol) in 5 mL of dichloromethane, was added $NaHCO_3$ (37 mg, 0.44 mol). The solution was cooled to 0°C and 3-chloroperbenzoic acid (*m*-CPBA, 76 mg, 0.44 mmol) was added with vigorous stirring. The color of the mixture turned from magenta to orange-yellow within 10 min. The reaction was warmed up to room temperature and furthered stirred for 2 hours and then purified by aluminum oxide (300-400 mesh) column chromatography (dichloromethane: methanol = 20:1) to afford a yellow color powder (75 mg, 77%).

^1H NMR (600 MHz, DMSO-d_6) δ 8.02 (d, *J* = 8.7 Hz, 4H), 7.94 (s, 2H), 7.92 – 7.87 (m, 8H), 7.16 (d, *J* = 8.7 Hz, 4H), 7.15 – 7.10 (m, 8H), 3.77 (dq, *J* = 11.6, 6.9 Hz, 8H), 3.45 – 3.40 (m, 8H), 0.97 (t, *J* = 7.0 Hz, 24H).

^{13}C NMR (151 MHz, DMSO-d_6) δ 153.84, 147.26, 146.58, 131.95, 131.63, 130.84, 128.15, 124.14, 123.89, 123.71, 66.40, 8.60.

HR-MS (TOF) m/z: $[M+H]^+$ Calc'd for $C_{58}H_{67}N_8O_4S^+$ 971.5000; Found 971.5000.

The preparation of TBTO NPs:

1 mg of TBTO was dissolved in 1 mL of THF and then added to 9 mL of ultrapure water containing 5 mg of DSPE-mPEG2000 in an ice bath, followed by sonication with a probe sonicator at an output power of 45% for 2 min (on/off cycle: 3 s/2 s). The resulting mixture was concentrated by ultrafiltration, washed with ultrapure water to remove THF, and then lyophilized and kept at 4°C for later use. For quantification of TBTO in nanoparticles, the lyophilized TBTO NPs were dissolved in DMSO and the loading content of TBTO was calculated using a pre-established calibration absorption curve of TBTO. The particle size, zeta potential, and morphology of TBTO NPs were examined by DLS and TEM.

Fe(II) sensing of TBTO NPs in vitro:

To 100 μL of TBTO NPs solution (50 μM) in PBS buffer (0.1 M, pH 7.4) in a 96-well plate was added 2 μL of metal ions solution in water (25 mM). The fluorescence intensity of TBTO at 600 nm (Ex: 450 nm) was monitored by a microplate reader (BioTek Synergy H1) before or after the treatment of metal ions for different time intervals. The working temperature of the microplate reader was set to 37°C.

The photothermal performance measurement of TBT and TBTO:

TBT and TBTO were dissolved in 200 μL of DMF-H_2O (1:1) at the concentration of 500 μM and then irradiated with a 660 nm laser at a power density of 0.5 W/cm^2 for 10 min. The temperature was recorded every 20 seconds.

Cell culture:

Human cervical cancer HeLa cell line was purchased from The Cell Bank of Type Culture Collection of Chinese Academy of Sciences and cultured in Dulbecco's Modified Eagle Medium (DMEM) with high glucose containing 10% Fetal Bovine Serum (FBS) and 1% antibiotics (penicillin-streptomycin) at 37°C in a humidified environment. Cells were culture in 5%CO_2 and 20% O_2 for normoxic conditions. Hypoxic cells were culture in 5% CO_2 and 0% O_2 in a sealed container (Mitsubishi, D-110) with an anaerobic gas generator (Mitsubishi, D-07), and the O_2 level was monitored by an anaero-indicator (Mitsubishi, D-66).

Cell cytotoxicity tests of TBTO and TBT:

HeLa cells were sub-culture into a 96 well plate with a density of 5×10^3 cells per well. After incubation at 37°C overnight for cell adherence, cells were treated with different concentrations of TBTO or TBT and then further cultured for 24 h. After that, cells were treated with MTT solution at a concentration of 0.5 mg/mL and then incubated for 4 h. MTT solution was discarded and 100 μL of DMSO was added per well, followed by a rigid shaking for 2 min to dissolve all the precipitates. The absorbance at 450 nm was measured by a microplate reader and the relative cell viability was calculated by the following equation: cell viability (%) = $(OD_{sample} - OD_{background)}$ / $(OD_{control} - OD_{background})$ × 100%.

Hypoxia responsiveness of TBTO NPs at a cellular level:

HeLa cells were sub-cultured into a 48 well plate with a density of 2.5×10^4 cells per well. After incubation at 37°C for 36 h to an 80% confluency, cells were transferred to either a standard atmosphere containing 20% oxygen (normoxic condition) or a sealed container with an anaerobic gas generator to keep the oxygen level lower than 0.1% (hypoxic condition). After pre-incubation for 12 h in the above conditions, cells were treated with different concentrations of TBTO NPs in an FBS-free medium and then a further incubation for 3 h in normoxic or hypoxic conditions. After that, the fluorescence intensities at 600 nm with an excitation of 450 nm of the cell culture supernatants were measured in a microplate reader. The FBS-free medium without TBTO NPs treatment was used as blank to deduct the background emission.

Tumor model establishment:

The male BALB/c nude mice (4~5 weeks) were obtained from Beijing Vital River Laboratory Animal Technology, and all the performances of in vivo experiments followed the protocols approved by the Administrative Committee on Animal Research in Shenzhen Graduate School, Peking University. A suspension of 5×10^6 HeLa cells in PBS (150 μL) was subcutaneously injected into each mouse to construct the tumor model. After about 7 days, mice with tumor volume at about 100 mm^3 were used for in vivo experiments.

In vivo NIR fluorescence imaging:

The hydrolyzed nanoparticles were dissolved in saline right before use.

Intratumoral injection: Two tumor-bearing mice were anesthetized with isoflurane and administered TBTO NPs and TBT NPs (50 μL, 1 mg/mL) via intratumoral injection, respectively. Immediately after that, these two mice were transferred to the small animal imaging system and imaged with excitation filters of 500 nm and 570 nm and corresponding emission filters of 720 and 740 nm. Data were processed using Living Image software (Version 4.5). ROIs with the equal area were drawn in the tumor site of each image, and average radiant efficiency in each ROIs was measured.

Tail intravenous injection: Two tumor-bearing mice were administered TBTO NPs via tail intravenous injection (200 μL, 1 mg/mL). At different time points post-injection, mice were anesthetized with isoflurane and imaged with a small animal imaging system with an excitation filter of 570 nm and corresponding emission filters of 720 and 740 nm. After 24 h, mice were sacrificed; the organs and tumors were imaged using the same filter as above. Data were

processed using Living Image software (Version 4.5). ROIs with the equal area were drawn in the tumor site of each image, and average radiant efficiency in each ROIs was measured.

Ex vivo colocalization with commercial hypoxia imaging kit

Three tumor-bearing mice were administered TBTO NPs via tail intravenous injection (200 μL, 1 mg/mL in saline). After normal feeding for 24 h, mice were then administered pimonidazole HCl (Hypoxyprobe-1, 100 μL, 2.8 mg/mL in saline) via tail intravenous injection and then sacrificed after 2 h. Tumors were harvested and then immunofluorescence staining in formalin-fixed and paraffin-embedded tissue sections was performed using FITC-conjugated anti-pimonidazole mouse IgG_1 monoclonal antibody (FITC-MAb1). Fluorescence images were acquired using CLSM with a tile mode to stitch 25 images to a large field of view.

In vitro PA imaging in tissue-mimicking phantoms:

Tissue phantoms were prepared referring to a literature reported method (Knox et al., 2018). In brief, agarose (4 g) was suspended in a solution of 2% milk (2 mL) and distilled water (78 mL) and then heated in a microwave oven for 1 min. The obtained viscous gel was transferred to a 50 mL centrifuge tube with a FEP tube (2 mm inner diameter, 10 cm long, sealed with cotton at the bottom) inside. After cooling for 3 h, the solidified phantom was removed from the centrifuge tube and cut with a knife so that the phantom was around 2 cm thick with the FEP tube in the middle. TBTO NPs or TBT NPs aqueous solution was filled in the FEP tube using a syringe with a long needle and then imaged with Vevo LAZR-X.

In vivo PA imaging:

The hydrolyzed nanoparticles were dissolved in saline right before use. Mice were administered TBTO NPs via tail intravenous injection (200 μL, 1 mg/mL) or intratumoral injection (200 μL, 1 mg/mL). After different time intervals post-injection, mice were then anesthetized with isoflurane, and images were acquired using the Step and Shoot mode with 100 angles and 15 pulses per angle. Mice were then sacrificed at different time points post-injection: 1 day, 2 days, and 3 days to check the bioelimination in different organs and tumors. Data were analyzed using Vevo LAB 3.2.0 software. Thick slab processing was used to visualize accumulated signals over 12 mm.

SUPPLEMENTAL REFERENCES

Tancini, F., Wu, Y.-L., Schweizer, W.B., Gisselbrecht, J.-P., Boudon, C., Jarowski, P.D., Beels, M.T., Biaggio, I., and Diederich, F. (2012). 1,1-Dicyano-4-[4-(diethylamino)phenyl]buta-1,3-dienes: structure–property relationships. Eur. J. Org. Chem. *2012*, 2756-2765.
